# Eukaryotic large nucleo-cytoplasmic DNA viruses: Clusters of orthologous genes and reconstruction of viral genome evolution

**DOI:** 10.1186/1743-422X-6-223

**Published:** 2009-12-17

**Authors:** Natalya Yutin, Yuri I Wolf, Didier Raoult, Eugene V Koonin

**Affiliations:** 1 National Center for Biotechnology Information, National Library of Medicine, National Institutes of Health, Bethesda, MD 20894, USA; 2 URMITE, Centre National de la Recherche Scientifique UMR IRD 6236, Faculté de Médecine, Université de la Méditerranée, 27 Boulevard Jean Moulin, 13385 Marseille Cedex 5, France

## Abstract

**Background:**

The Nucleo-Cytoplasmic Large DNA Viruses (NCLDV) comprise an apparently monophyletic class of viruses that infect a broad variety of eukaryotic hosts. Recent progress in isolation of new viruses and genome sequencing resulted in a substantial expansion of the NCLDV diversity, resulting in additional opportunities for comparative genomic analysis, and a demand for a comprehensive classification of viral genes.

**Results:**

A comprehensive comparison of the protein sequences encoded in the genomes of 45 NCLDV belonging to 6 families was performed in order to delineate cluster of orthologous viral genes. Using previously developed computational methods for orthology identification, 1445 Nucleo-Cytoplasmic Virus Orthologous Groups (NCVOGs) were identified of which 177 are represented in more than one NCLDV family. The NCVOGs were manually curated and annotated and can be used as a computational platform for functional annotation and evolutionary analysis of new NCLDV genomes. A maximum-likelihood reconstruction of the NCLDV evolution yielded a set of 47 conserved genes that were probably present in the genome of the common ancestor of this class of eukaryotic viruses. This reconstructed ancestral gene set is robust to the parameters of the reconstruction procedure and so is likely to accurately reflect the gene core of the ancestral NCLDV, indicating that this virus encoded a complex machinery of replication, expression and morphogenesis that made it relatively independent from host cell functions.

**Conclusions:**

The NCVOGs are a flexible and expandable platform for genome analysis and functional annotation of newly characterized NCLDV. Evolutionary reconstructions employing NCVOGs point to complex ancestral viruses.

## Introduction

Viruses span approximately 3 orders of magnitude (~10^3 ^to ~10^6 ^nucleotides) in genome size and show tremendous diversity of virion architecture, size and complexity [1-3]. Highly diverse viruses share homologous "hallmark genes" encoding some of the key proteins involved in genome replication and virion structure formation [4]. However, no gene is common to all viruses, so there is no evidence of a monophyletic origin of all viruses, at least, not within the traditional concept of monophyly. Nevertheless, large groups of viruses infecting diverse hosts do appear to be monophyletic as indicated by the conservation of sets of genes encoding proteins responsible for most of the functions essential for virus reproduction. One of the most expansive, apparently monophyletic divisions of viruses consists of at least 6 families of eukaryotic viruses with large DNA genomes including Poxviridae, an expansive viral family that includes major pathogens of humans and other mammals. These viruses infect animals and diverse unicellular eukaryotes, and replicate either exclusively in the cytoplasm of the host cells, or possess both cytoplasmic and nuclear stages in their life cycle (Table 1). These viral families have been collectively designated Nucleo-Cytoplasmic Large DNA Viruses (NCLDV) [5,6].

**Table 1:** The 6 NCLDV families used for the NCVOG construction

Virus family	Host range	Genome size range, kb	Replication site
**Phycodnaviridae**	Green algae; algal symbionts of paramecia and hydras	150-400	Nucleus and cytoplasm
**Poxviridae**	Animals: insects, reptiles, birds, mammals	130-380	Cytoplasm
**Asfarviridae**	Mammals	170	Cytoplasm
**Asco- and Iridoviridae**	Invertebrates and non-mammalian vertebrates	100-220	
**Ascoviridae**	Insects, mainly, Noctuids	150-190	Nucleus and cytoplasm
**Iridoviridae**	Insects, cold-blooded vertebrates	100-220	Nucleus and cytoplasm
**Mimiviridae**	Acanthamoeba	1,180	Cytoplasm
**Marseillevirus**	Acanthamoeba	370	Nucleus and cytoplasm(?)

Generally, the NCLDV do not show strong dependence on the host replication or transcription systems for completing their replication [7]. This relative independence of the viruses from the host cells is consistent with the fact that all these viruses encode several conserved proteins that mediate most of the processes essential for viral reproduction. These key proteins include DNA polymerases, helicases, and DNA clamps responsible for DNA replication, Holliday junction resolvases and topoisomerases involved in genome DNA manipulation and processing, transcription factors that function in transcription initiation and elongation, ATPase pumps for DNA packaging, and chaperones involved in the capsid assembly [5,6]. Although only 9 genes were found to be conserved in all NCLDV (with sequenced genomes), a considerable number of additional genes are shared by diverse viruses from multiple families. An evolutionary reconstruction using a parsimony approach mapped approximately 40 genes to the putative common ancestor of the NCLDV [6]. Thus, it appears that the ancestral NCLDV already was a complex virus that generally resembled the extant members of this group and was capable of relatively independent reproduction in the cytoplasm of the host cells, the exact identity of the host notwithstanding [6,8].

The NCLDV share some of the virus hallmark genes [4] with other large DNA viruses such as herpesviruses and baculoviruses. Examples of such shared hallmark genes include the B-family DNA polymerases, DNA primases, and Superfamily 2 helicases related to herpesvirus origin-binding protein UL9. However, most of the NCLDV share a considerable number of additional genes to the exclusion of other large DNA viruses of eukaryotes. Cases in point include the Superfamily 3 helicase (typically, fused with primase in NCLDV), the packaging ATPase, the disulfide oxidoreductase involved in virion morphogenesis, and more. The existence of these signature NCLDV genes, despite the notable connectivity of the virus world, justifies the classification of the NCLDV as distinct, monophyletic class of viruses [5,6].

In the last few years, the NCLDV attracted much new attention owing, primarily, to the discovery and genome sequencing of the giant Mimivirus that was isolated from Acanthamoeba. At ~1.2 Mb, the Mimivirus and the closely related Mamavirus possess by far the largest genomes of all known viruses [9-13]. These viruses encompass the full complement of conserved NCLDV genes but also possess numerous genes homologous to genes of cellular organisms including several encoding translation system components. The unexpected discovery of these genes in the mimivirus led to speculation on the origin of the giant viruses from a putative "fourth domain of cellular life" by genome degradation [14]. However, comparison of the mimivirus gene repertoire with those of other NCLDV combined with phylogenetic analysis of both conserved NCLDV genes and the homologs of host genes encoded by the mimivirus indicate that the Mimivirus is a bona fide NCLDV and appears to be related to phycodnaviruses and iridoviruses [6]. The homologs of genes of cellular organisms, in all likelihood, were acquired in the course of evolution of the mimivirus lineage, probably, from a variety of distinct cellular sources; the same process of horizontal acquisition of cellular genes occurred, on a smaller scale, in all other families of the NCLDV [6,8,15-18].

Very recently, another giant virus, named Marseillevirus, was isolated from Acanthamoeba. Genome analysis of Marseillevirus indicated that it represents a putative novel family of NCLDV that appears to be distantly related to iridoviruses and ascoviruses [19]. In addition, comparative-genomic analysis revealed probable gene exchange between Marseillevirus and Mimiviruses, emphasizing the role of amoeba as a "melting pot" of NCLDV evolution.

An interesting new perspective on the NCLDV emerged from the rapid progress of metagenomic studies. It turns out that the DNA samples from the Global Ocean Survey contain numerous sequences homologous to genes of all known NCLDV families, except for *Poxviridae *and *Ascoviridae*, and possibly, representatives of new families as well [19-23]. Thus, there seems to exist a considerable unexplored diversity of NCLDV that most likely infect various unicellular eukaryotes but, possibly, also marine invertebrates [24].

As the number of available viral genomes quickly grows, both challenges and the potential of comparative and evolutionary genomics of the NCLDV increase. A pre-requisite of an informative comparative-genomic study of any group of organisms is an accurate delineation of the sets of orthologous genes, that is, genes that evolved from the same gene in the genome of the last common ancestor of the compared genomes [25,26]. Accurate identification of clusters of orthologous (COGs) is essential both for functional annotation of uncharacterized genes and for evolutionary reconstructions. The COG analysis has been initially applied in a comprehensive manner, to all then available genomes of archaea, bacteria and unicellular eukaryotes [27,28], but subsequently, with the exponential growth of the collections of sequenced genomes, it became more realistic to derive COGs for compact taxa such as archaea or cyanobacteria [29,30]. The NCLDV, with their large (on the virus scale) genomes consisting of genes with different degrees of evolutionary conservation, are in need of and amenable to the same approach. Here we describe the construction of clusters of orthologous genes for the NCLDV which we abbreviate as NCVOGs (Nucleo-Cytoplasmic Virus Orthologous Genes) which we intend as a platform for the functional and evolutionary analysis of new NCLDV genomes. We also report some patterns of evolution of the NCLDV that can be inferred from a preliminary analysis of the NCVOGs.

## Results and Discussion

### Clusters of orthologous genes for the NCLDV (NCVOGs)

**Figure 1:**
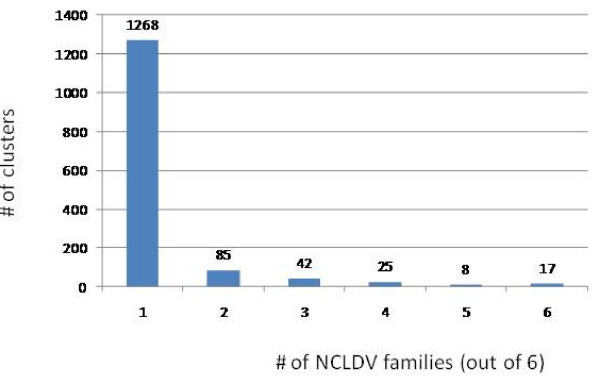
**Distribution of the number of NCLDV families represented in NCVOGs**.

**Figure 2:**
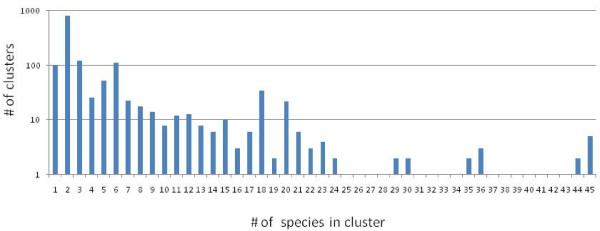
**Distribution of the number of NCLDV species represented in NCVOGs**.

In this works, we analyzed the annotated proteins encoded in 45 NCLDV proteomes from 6 viral families (Tables 1 and Additional file 1). These viral proteins were partitioned into clusters of likely orthologs using a modified COG procedure (Ref. [30]; see Methods for details). All clusters were manually edited and annotated using the results of RPS-BLAST and PSI-BLAST searches for the constituent proteins. Of the 11,468 (predicted) proteins encoded in the 45 NCLDV genomes, 9,261 were included into 1,445 clusters of probable orthologs (NCVOGs). The overwhelming majority of the NCVOGs (1,268) are family-specific (that is, include proteins from viruses of only one family) whereas the remaining 177 NCVOGs included proteins from two or more NCLDV families (Figure 1). The distribution of the NCVOGs by the number of viral species showed a qualitatively similar pattern where the most abundant class included two species (thanks to closely related pairs of viruses with very large genomes such as the mimivirus and the mamavirus) and additional peaks corresponded to large viral families such as Poxviridae or Phycodnaviridae with 6 (selected) representatives (Figure 2).

**Figure 3:**
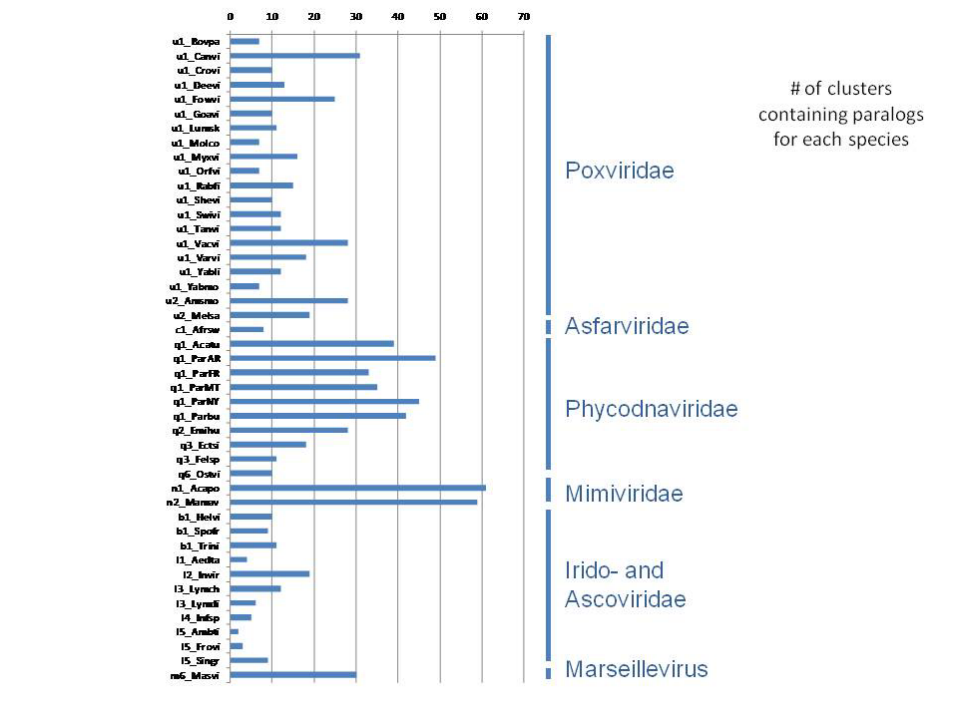
**Numbers of NCVOGs that include paralogs in each of the analyzed viruses**.

**Figure 4:**
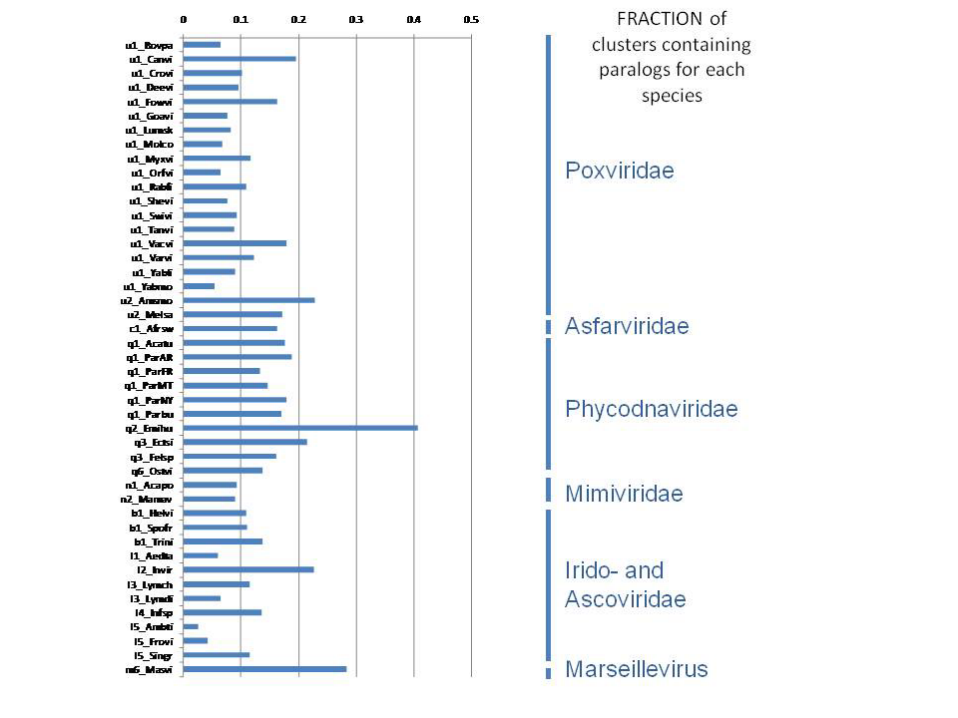
**Fractions of NCVOGs that include paralogs in each of the analyzed viruses**.

**Figure 5:**
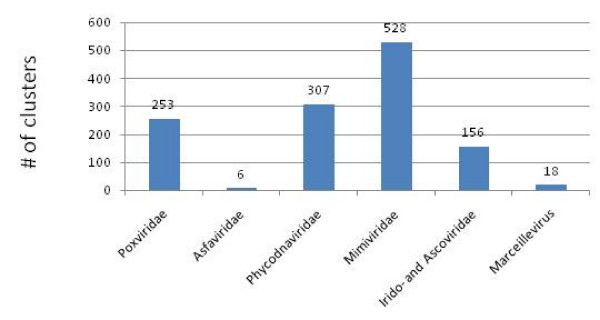
**Distribution of the 1268 family-specific NCVOGs among the 6 NCLDV families**.

Many of the NCVOGs include multiple paralogs from the same virus that were recognized by the clustering procedure and assigned to the same cluster. As expected, paralogs were most common and numerous in viruses with the largest genomes, namely, mimiviruses and phycodnaviruses (Figures 3, 4). In the same vein, the mimiviruses and the phycodnaviruses made the dominant contribution to the 1,268 family-specific NCVOGs (Figure 5).

**Figure 6:**
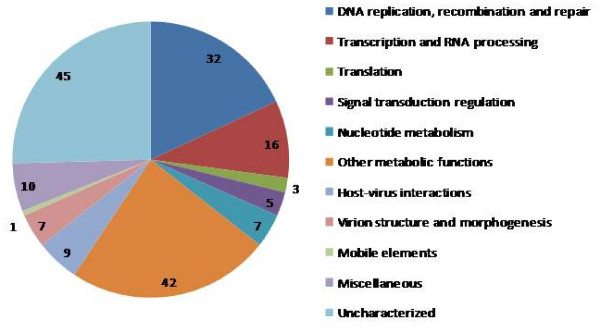
**Functional classification of the 177 NCVOGs that include two or more NCLDV families**.

The 177 multifamily NCVOGs were annotated with respect to the known or predicted functions and assigned to several broad functional classes (Figure 6 and Additional File 1). Notably, the widespread NCVOGs consist of genes that encode proteins involved in key functions of viral replication and morphogenesis as is typical of viral hallmark genes (Additional File 1). It is also of note that among the 177 widespread NCVOGs there are virtually none without an assigned function (at least in general terms; Additional File 1). Thus, transfer of functional information from experimentally characterized viral genes to uncharacterized orthologs in other viruses yields a fairly complete compendium of the core NCLDV functions.

### Phylogenies of the core proteins of the NCLDV

As the number of genomes of cellular life available for comparative analysis increases, the set of universal genes, which comprised a small fraction of the genes even in the original COG analysis [28], continues to shrink [31,32]; in large part, this is a consequence of non-orthologous displacement whereby the same indispensable function is mediated by unrelated genes in different life forms [33]. Non-orthologous gene displacement as well as lineage-specific gene loss seem to be important in the evolution of the NCLDV as well, the result being that only a few genes are conserved in all viruses of this class. In the present analysis, only 5 NCVOGs included proteins from all 45 analyzed viruses, namely, the major capsid protein (orthologs of vaccinia virus D13 protein), primase-helicase (VV D5), Family B DNA polymerase (VV E9), packaging ATPase (VV A32), and transcription factor (VV A2). Given the previous conclusions on the origin of the NCLDV from a single ancestral virus [5,6], we sought to reconstruct the phylogeny of the NCLDV by analyzing the phylogenetic trees of these highly universal proteins as well as additional highly conserved proteins. The capsid protein is not suitable for reconstructing NCLDV phylogeny: the sequences of the capsid protein ortholog in poxviruses (VV D13) are extremely divergent, resulting in low information content of the alignment, and other viruses encode multiple paralogs of the capsid protein). The remaining 4 conserved proteins yielded phylogenetic trees with somewhat conflicting topologies (Additional File 2). Assuming that the conflicts were caused by tree construction artifacts rather than genuinely different histories of different core gene of the NCLDV, we employed the consensus tree approach (see Methods for details) to reconstruct the putative NCLDV phylogeny using 10 trees of genes that are represented in all or nearly all of the NCLDV. Specifically, the phylogenies of the following 10 conserved genes contributed to the consensus tree: Superfamily II helicase, A2L-like transcription factor, RNA polymerase A subunit, RNA polymerase B subunit, mRNA capping enzyme, A32-like packaging ATPase, small subunit of ribonucleotide reductase, Myristylated envelope protein, primase-helicase, and DNA polymerase (See Additional File 2).

**Figure 7:**
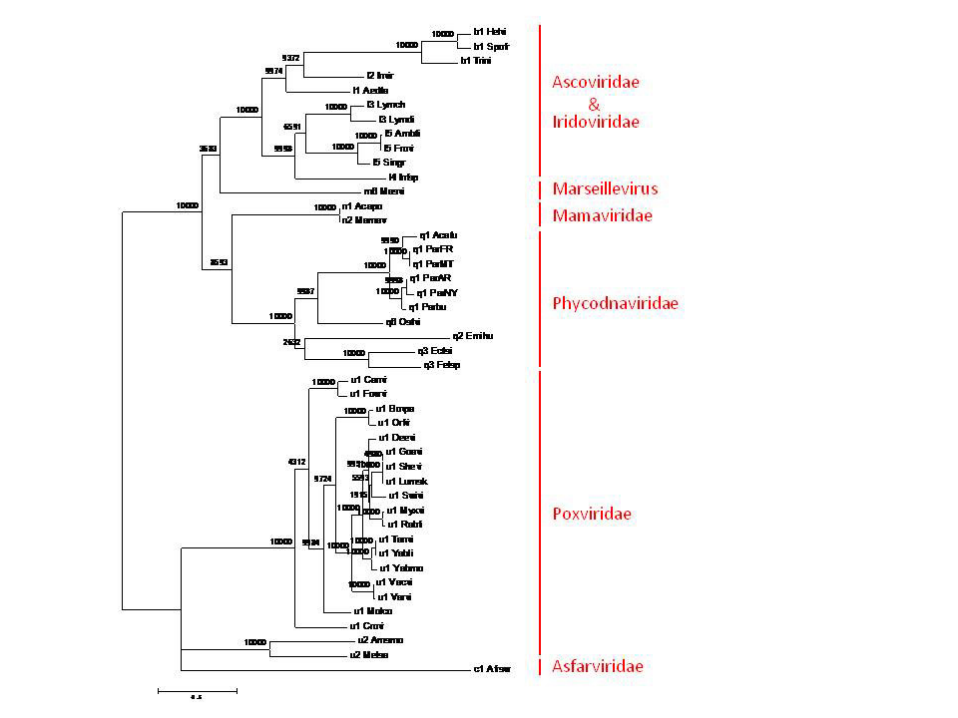
**The consensus phylogenetic tree of the NCLDV**. The Expected Likelihood Weights (1,000 replications) are indicated for each ancestral node as percentage points. The topology of the tree was derived as the consensus of the tree topologies for the following 10 (nearly) universal NCVOGs: Superfamily II helicase (NCVOG0076), A2L-like transcription factor (NCVOG0262), RNA polymerase α subunit (NCVOG0274), RNA polymerase β subunit (NCVOG0271), mRNA capping enzyme, A32-like packaging ATPase (NCVOG0249), small subunit of ribonucleotide reductase (NCVOG0276), Myristylated envelope protein (NCVOG0211), primase-helicase (NCVOG0023), and DNA polymerase (NCVOG0038) (See Additional File 2). The branch lengths and ELW values (shown as percentage points) are from a tree that was constructed from a concatenated alignment of 4 universal proteins (primase-helicase, DNA polymerase, packaging ATPase, and A2L-like transcription factor).

In the best supported consensus tree topology, the recently discovered Marseillevirus clustered with irido- and ascoviruses (the latter were confidently placed inside the Iridoviridae), albeit with a low confidence; mimiviruses clustered with phycodnaviruses; and poxviruses grouped with asfarviruses (Figure 7). Of the 10 trees that contributed to the consensus tree, 5 displayed the same topology, at the level of major branches (viral families), as the consensus tree and 3 were compatible with the consensus topology (Approximately Unbiased (AU) test [34] p-value > 0.05). The trees of the DNA polymerase and primase-helicase showed significant differences (p < 0.05) from the consensus (see Additional File 2) according to the AU test. In the DNA polymerase tree, phycodnaviruses confidently grouped with the Irido-Marseillevirus branch, in contrast to the phycodna-mimi clade in the consensus tree. The primase-helicase tree was the "worst" in terms of conformity to the consensus, with the unusual but strongly supported Mimi-Irido-Marseille clade and moderately supported joining of asfarviruses to that branch (compare the trees in Figure 7 and Additional File 2). Given the propagation of mimiviruses and Marseillevirus in the host (Acanthamoeba) [19], the recent isolation of an asfarvirus from a dinoflagellate [35], and indications from metagenomics that iridoviruses might infect marine unicellular eukaryotes as well [21,23], horizontal exchange of these essential genes among viruses from different families cannot be ruled out. Further investigation of this intriguing possibility requires deeper genomic sampling of NCLDV and a comprehensive phylogenetic analysis (see also below).

We further constructed a different type of tree for the NCLDV, one that was based on the comparison of gene repertoires, more specifically, the patterns of representation of viruses in NCVOGs, also known as phyletic patterns [36]. The trees were produced from the 15 × 1445 matrix of subfamily-level phyletic patterns using the neighbor-joining tree reconstruction method and 4 different methods for distance calculation (see Methods for details and Additional File 3). The topologies of these gene content trees were generally compatible with that of the consensus tree (Figure 3), indicating that the evolution of the gene repertoire of the NCLDV, largely, mirrored the evolution of the conserved core genes. However, there was one notable exception to this congruence: in 3 of the 4 gene content trees, Marseillevirus clustered with the Mimiviridae. This similarity of gene repertoires, most probably, stems from the reproduction of these viruses in the same host (Acanthamoeba) where the viruses repeatedly exchanged genes during their evolution [19].

### Conserved genes and reconstruction of the evolution of the NCLDV gene repertoire

**Figure 8:**
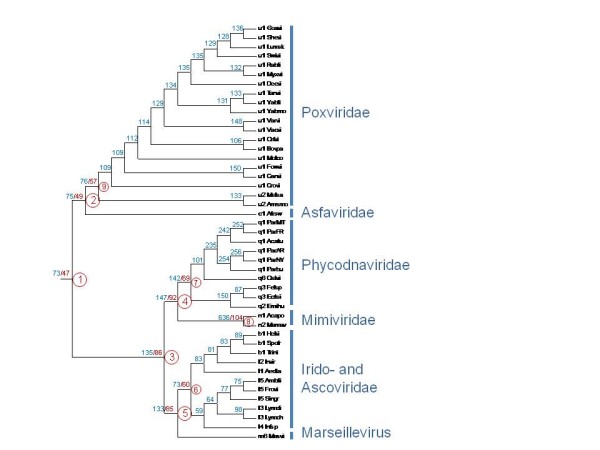
**Reconstruction of the ancestral NCLDV gene sets**. The inferred numbers of genes present in each internal node are shown in blue. Numbers of NCVOGs present with the likelihood greater than 0.9 for 9 deepest nodes (numbered) are shown in red. For the complete list of these NCVOGs, see Additional File 4. The tree from Figure 3 was used as a guide for the reconstruction.

**Figure 9:**
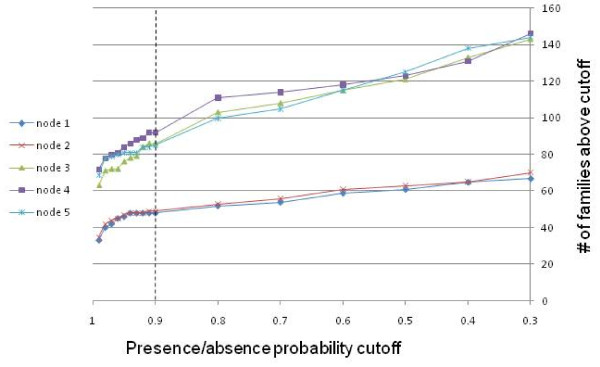
**The size of reconstructed ancestral gene sets depending on the likelihood threshold**.

We employed the consensus tree of the NCLDV (Figure 7) to reconstruct the core gene repertoires of ancestral viruses and gene loss and gain events during the evolution of the NCLDV using the maximum-likelihood approach developed by Csuros and Miklos [37]. Using a likelihood cut-off of 0.9, we found that 47 genes mapped to the common ancestor of the NCLDV and reconstructed progressively increasing gene repertoires for other ancestral viruses (Figure 8, Additional Files 4 and 5). The ancestral gene repertoires were relatively insensitive to the likelihood cut-off (Figure 9), an observation that seems to support the reliability of the reconstruction. Undoubtedly, these are conservative reconstructions because it is not feasible to assign to ancestral forms genes that survived in only one of the progeny lineages let alone those that were lost in all extant lineages. Nevertheless, the reconstructed gene repertoire suggests that the common ancestor of all known NCLDV possessed all the core functions characteristic of this class of viruses. These functions include the basal machineries for replication, transcription and transcript processing (such as the capping and decapping enzymes), enzymes required for DNA precursor synthesis (thymidine kinase and thymidylate kinase), the two major virion proteins, the central enzymes of virion morphogenesis (protease and disulfide oxidoreductase), and even some proteins implicated in virus-cell interaction such as a RING-finger ubiquitin ligase subunit (see Additional File 4). A caveat is that some of these genes might have spread among the NCLDV via extensive between-virus gene transfer.

Some of the core functions are prone to non-orthologous displacement among the NCLDV, sometimes showing complex evolutionary patterns. A case in point is the DNA ligase that is an essential activity for DNA replication. The previous reconstruction of the ancestral NCLDV gene repertoire tentatively identified the ATP-dependent ligase as an ancestral NCLDV gene [5,6]. However, entomopoxviruses, mimiviruses, and some of the iridoviruses lack the ATP-dependent ligase and instead encode a distinct NAD-dependent ligase (of apparent bacterial origin) (see Additional Files 1 and 4). Furthermore, some poxviruses, such as Molluscum Contagiosum virus [38], encode no ligase at all, apparently, as a result of lineage-specific gene loss; in such cases, this essential replication function is probably supplied by a host ligase. The present maximum-likelihood reconstruction mapped both ligases to the ancestral NCLDV genome. However, phylogenetic analysis of the ATP-dependent and NAD-dependent ligases yielded an unexpected result: the NAD-dependent ligases of the NCLDV formed an unequivocally supported clade whereas the ATP-dependent showed different phylogenetic affinities [39]. The conclusion, perhaps, a counterintuitive one is that the NAD-dependent ligase, of bacteriophage or bacterial origin, is the ancestral NCLDV gene that was repeatedly displaced by ATP-dependent ligases in different viral lineages [39]. These findings reveal inherent limitations of reconstructions of ancestral gene repertoires based on patterns of gene presence-absence.

Owing to non-orthologous displacement, some of genes encoding (nearly) essential functions might not have made it to the reconstructed ancestral gene repertoire. An interesting potential case of such missing function is that of phospholipase that is likely to be required for NCLDV morphogenesis as well as for the escape of the virus from the host phagosomes. A large subset of the NCLDV including mimiviruses, Marseillevirus, and some phycodnaviruses and iridovirsues encode a patatin-family phospholipase (Additional File 1) that has been implicated in the pathogen-host interaction of intracellular bacterial parasites such as *Legionella *[40]. In poxviruses, this phospholipase is missing but there are one or two paralogous genes encoding a distinct enzyme of the phospholipase D family which is part of the virus envelope [41] and is involved in the formation of virus-specific vesicles in infected cells [42]. It seems plausible that the ancestral NCLDV encoded the patatin-like phospholipase that was subsequently displaced by the unrelated phospholipase D-like enzyme in poxviruses. Similar patterns of non-orthologous gene displacement are likely to involve additional NCLDV genes, emphasizing the inevitable conservative character of the evolutionary reconstruction.

The results of the evolutionary reconstruction indicate that the common ancestor of the NCLDV already was a bona fide virus of this class and, in particular, possessed the same degree of independence of the host cell functions as the extant NCLDV. The NCLDV infect diverse eukaryotes including a wide range of unicellular forms, and moreover, remarkable diversity of the hosts is seen even within some of the NCLDV branches; the relationship between irido-ascoviruses infecting animals and Marseillevirus that reproduces in Acanthamoeba is a case in point (Figures 3 and 4). Thus, it appears most likely that this full-fledged ancestral NCLDV evolved at an early stage of eukaryotic evolution, prior to the divergence of the eukaryotic supergroups, and that the radiation of the branches of the NCLDV was a very early event as well. It is tempting to speculate that this initial radiation of the NCLDV occurred as a "Big Bang-like" event concomitantly with eukaryogenesis [4], a model similar to that recently elaborated for a completely different group of eukaryotic viruses, the picorna-like superfamily of RNA viruses [43].

The actual genome size and complexity of the ancestral NCLDV is a wide-open question. Clearly, the 47 genes mapped to the ancestral genome in the present reconstruction comprise only the core of most highly conserved, essential viral genes involved in key functions. Given that the ancestral NCLDVs undoubtedly reproduced in unicellular eukaryotes, and this type of host supports the propagation of extant giant viruses, such as the mimiviruses [13,24], it cannot be ruled out that already at an early stage of evolution the ancestral NCLDV genome grew highly complex. Thus, the common ancestor of all extant NCLDV even might have been a giant virus.

## Conclusions

The goal of this work was to classify the genes from the growing collection of the NCLDV genomes into clusters of probable orthologs and in-paralogs in order to facilitate annotation of newly sequenced viral genomes and analysis of viral evolution. It is our hope that the curated set of NCVOGs will serve these purposes, in particular, with respect to new giant viruses that undoubtedly will be isolated from unicellular eukaryotes in the nearest future. The comparative analysis of the NCLDV genes showed that only 177 of the 1445 NCVOGs include representatives from more than one virus family. An even smaller set of 47 conserved genes was mapped to the common ancestor of the NCLDV by the maximum-likelihood reconstruction. This reconstructed ancestral gene set is robust to the parameters of the reconstruction procedure and does not dramatically differ from the ancestral gene set reconstructed previously on a smaller collection of viral genomes and using a simpler, parsimony method [6]. In particular, the inclusion of representatives of two additional virus families, the *Ascoviridae *and the putative new family represented by the Marseillevirus, did not result in an erosion of the reconstructed ancestral gene set. However, detailed phylogenetic analysis can lead to some revisions of the ancestral gene set as illustrated by the case of ATP-dependent and NAD-dependent DNA ligases. These caveats notwithstanding, it seems that the reconstruction reflects the gene core of the ancestral NCLDV with a reasonable accuracy and indicates that this virus encoded a complex machinery of replication, expression and morphogenesis that made it relatively independent from host cell functions.

## Methods

### Construction of the NCVOGs

For the construction of the NCVOGs, we used 45 annotated protein sets of Nucleo-Cytoplasmic Large DNA viruses (NCLDV) (see Additional File 6; 5 closely related Orthopoxviruses were not included).

The conceptual proteomes of Marseillevirus and Mamavirus were obtained by translation of the respective genomic nucleotide sequences using the GeneMark software [44]. Other proteomes were downloaded from GenBank http://www.ncbi.nlm.nih.gov/. The complete data set consisted of 11,219 protein sequences. The procedure of NCVOG construction involved the following steps.

1) Ankyrin repeat-containing proteins were the most abundant proteins in the data set (~400 proteins, or 3.5% of the data set). Owing to the low sequence complexity of these proteins, they produced large number of false-positive hits during similarity searches. These proteins were removed from the data set prior to clustering.

2) All-against-all BLASTP [45] search and initial clustering was performed using a modified COG construction algorithm [30]. At this step, 7,804 proteins were grouped into 1,571 clusters.

3) Multiple alignments of the initial cluster members were constructed using the MUSCLE program [46]. The alignments were used to construct position-specific scoring matrices (PSSM) for a PSI-BLAST search against the NCLDV protein dataset. Hits with e-values below 0.01 were reviewed, and clusters were merged when appropriate.

4) Clusters were further manually checked and edited using BLASTCLUST http://www.ncbi.nlm.nih.gov/IEB/ToolBox/C_DOC/lxr/source/doc/blast/blastclust.html and RPS-BLAST [47]. As a result of these refinement procedures, 1,445 NCVOGs consisting of 9,261 proteins were obtained.

5) The NCVOGs were manually annotated on the basis of RPS-BLAST and PSI-BLAST hits of cluster members.

The NCVOGs are available at ftp://ftp.ncbi.nih.gov/pub/wolf/COGs/NCVOG/.

### Multiple alignment and phylogenetic tree construction

The sequences for phylogenetic analysis were aligned using MUSCLE [46]. Poorly conserved positions and positions including gaps in more than one-third of the sequences were removed prior to tree computation.

Maximum Likelihood trees (ML) were constructed using TreeFinder [48], with the estimated site rates heterogeneity and the WAG (Whelan and Goldman) substitution model [49]. The Expected-Likelihood Weights (ELW) of 1,000 local rearrangements were used as confidence values of TreeFinder tree branches. Phylogenetic tree topologies were compared using the Approximately Unbiased (AU) test [34].

### Consensus trees

#### Relationships between viral families

At the first step, relationships between the 6 NCLDV families (*Poxviridae*, *Asfarviridae*, *Irido*- and *Ascoviridae*, *Mimiviridae*, *Phycodnaviridae*, and *Marseillevirus*) were resolved by analysis of the 49 NCVOGs that included representatives of at least 4 of the 6 families (49 clusters; Additional File 1). For these NCVOGs, ML trees were built from protein sequence alignments. Only 10 out of 49 NCVOGs produced alignments and trees deemed suitable for further analysis; the rest were discarded for one of the following reasons: there were too few (less than two) representatives from one or more families; there were too few (less than 100) conserved positions; one or more viral families appeared non-monophyletic. All 105 possible topologies corresponding to the relationships between 6 viral families were compared to the topologies of the 10 trees of individual conserved genes using the TOPD software [50]. The consensus topology (Figure 7) was supported by 5 of the 10 NCVOGs (HelicaseII, A2L-like transcription factor (Pox_VLTF3), RNA polymerase A, RNA polymerase B, mRNA capping enzyme) and was accordingly chosen as the family-level consensus topology.

#### Relationships between species

At the second step, topologies inside Irido-, Phycodna-, and Poxviridae were resolved as follows. NCVOGs with high representation of family members (19 NCVOGs for Iridoviridae, 12 for Phycodnaviridae and 43 for Poxviridae) were used to build ML trees from protein sequence alignments. Two to four orthologs from other NCLDV families or cellular homologs were used as the outgroup for Iridoviridae and Phycodnaviridae; Poxviridae trees were rooted between Chordopoxvirinae and Entomopoxvirinae. After discarding poorly conserved families (less than 100 conserved positions) 17, 6 and 42 trees remained for Iridoviridae, Phycodnaviridae and Poxviridae, respectively. The topology most compatible with the rest of the family-specific trees was identified using the Bootsplit method [51] and used as the consensus.

#### Full consensus tree

The topologies obtained at the first and second steps were combined in a consensus tree. A concatenated alignment of four proteins present in all 45 species (D5_helicase_primase, DNApol_B, Pox_A32_pfam04665 and Pox_VLTF3) was used to calculate branch lengths and ELW values for the consensus tree using TreeFinder [48].

### Neighbor-Joining gene content trees from phyletic patterns

Gene content trees for 15 NCLDV subfamilies were constructed as follows. Original 45 × 1445 binary presence/absence matrix (genome-level phyletic patterns) was converted into the 15 × 1445 subfamily-level presence/absence matrix by applying the logical OR operation within a subfamily (i.e. a subfamily registers a presence of an NCVOG if at least one genome of this subfamily has a protein from this NCVOG). For each pair of subfamilies the number of NCVOGs present in each of them (*N*_1 _and *N*_2_) as well as the number of NCVOGs present in both (*N*_U_) were computed. Then a gene content similarity measure (*s*) was calculated as either *s *= *N*_U_/min(*N*_1_, *N*_2_) or *s *= *N*_U_/sqrt(*N*_1 _× *N*_2_) and converted to a distance measure (*d*) as either *d *= 1-*s *or *d *= -ln(*s*). Neighbor-joining trees were constructed from the distance matrices using the NEIGHBOR program of Phylip 3.66 [52]. Bootstrap values were obtained by 100 resamplings of the subfamily-level phyletic patterns.

### Reconstruction of gene gain and loss events during the evolution of NCLDVs

Reconstruction of gene content evolution in the history of the NCLDV was performed using Count software http://www.iro.umontreal.ca/~csuros/gene_content/count.html[37,53]. The software infers gene gain, loss and duplication rates on the branches of the species tree from the 45 × 1445 matrix of genome-level phyletic patterns using the likelihood maximization based on a phylogenetic birth-and-death model. The consensus tree (Figure 3) was used as the guide topology; the model assumed the Poisson family size distribution at the tree root and uniform gain, loss and duplication rates. Inferred model parameters include probabilities for each NCVOG to be present in each of the ancestral nodes. The sum of these probabilities gives a relatively robust estimate of the ancestral genome size, whereas the specific list of the ancestral NCVOGs is a subject to much uncertainty because it might include multiple low-confidence families. Here we chose to report high-confidence (p > 0.9) genes as the likely candidates for the ancestral gene set.

## Competing interests

The authors declare that they have no competing interests.

## Authors' contributions

EVK designed the project; NY collected and analyzed data; YIW wrote software and analyzed data; DR and EVK wrote the manuscript that was read and approved by all authors

## Supplementary Material

Additional file 1 Functional classification of the 177 NCVOGs represented in two or more NCLDV families. Click here for file

Additional file 2 The ML trees for 10 (nearly) universal NCLDV proteins: D5-like helicase-primase (D5_helicase_primase); Family B DNA polymerase (DNApol_B); A32-like packaging ATPase (Pox_A32_pfam04665); A2L-like transcription factor (Pox_VLTF3); Ribonucleotide reductase, small subunit; RNA polymerase, α-subunit; RNA polymerase, β-subunit;superfamily II helicase; mRNA capping enzyme, large subunit; Myristylated envelope protein. Click here for file

Additional file 3 Neighbor-joining trees for 15 NCLDV subfamilies based on the patterns of presence/absence in the NCVOGs. Click here for file

Additional file 4 The reconstructed gene set for the common ancestor of the NCLDV. Click here for file

Additional file 5 Reconstructed gene sets for 9 internal nodes of the NCLDV tree. Click here for file

Additional file 6 The NCLDV genomes analyzed in this study. Click here for file

## References

[B1] Fields BN Howley PM Griffin DE Lamb RA Martin MA Roizman B Straus SE Knipe DM (eds.) Fields Virology 2001 New York: Lippincott Williams & Wilkins

[B2] Forterre P The origin of viruses and their possible roles in major evolutionary transitions Virus Res 2006 117 1 5 16 10.1016/j.virusres.2006.01.010 16476498

[B3] Raoult D Forterre P Redefining viruses: lessons from Mimivirus Nat Rev Microbiol 2008 6 4 315 319 10.1038/nrmicro1858 18311164

[B4] Koonin EV Senkevich TG Dolja VV The ancient Virus World and evolution of cells Biol Direct 2006 1 29 10.1186/1745-6150-1-29 16984643 PMC1594570

[B5] Iyer LM Aravind L Koonin EV Common origin of four diverse families of large eukaryotic DNA viruses J Virol 2001 75 23 11720 11734 10.1128/JVI.75.23.11720-11734.2001 11689653 PMC114758

[B6] Iyer LM Balaji S Koonin EV Aravind L Evolutionary genomics of nucleo-cytoplasmic large DNA viruses Virus Res 2006 117 1 156 184 10.1016/j.virusres.2006.01.009 16494962

[B7] Van Etten JL Unusual life style of giant chlorella viruses Annu Rev Genet 2003 37 153 195 10.1146/annurev.genet.37.110801.143915 14616059

[B8] Filee J Lateral gene transfer, lineage-specific gene expansion and the evolution of Nucleo Cytoplasmic Large DNA viruses J Invertebr Pathol 2009 101 3 169 171 10.1016/j.jip.2009.03.010 19457437

[B9] La Scola B Desnues C Pagnier I Robert C Barrassi L Fournous G Merchat M Suzan-Monti M Forterre P Koonin E Raoult D The virophage as a unique parasite of the giant mimivirus Nature 2008 455 7209 100 104 10.1038/nature07218 18690211

[B10] Raoult D Audic S Robert C Abergel C Renesto P Ogata H La Scola B Suzan M Claverie JM The 1.2-megabase genome sequence of Mimivirus Science 2004 306 5700 1344 1350 10.1126/science.1101485 15486256

[B11] Claverie JM Abergel C Mimivirus and its Virophage Annu Rev Genet 2009 43 49 66 19653859 10.1146/annurev-genet-102108-134255

[B12] Claverie JM Abergel C Ogata H Mimivirus Curr Top Microbiol Immunol 2009 328 89 121 full_text 19216436 10.1007/978-3-540-68618-7_3

[B13] Suzan-Monti M La Scola B Raoult D Genomic and evolutionary aspects of Mimivirus Virus Res 2006 117 1 145 155 10.1016/j.virusres.2005.07.011 16181700

[B14] Claverie JM Ogata H Audic S Abergel C Suhre K Fournier PE Mimivirus and the emerging concept of "giant" virus Virus Res 2006 117 1 133 144 10.1016/j.virusres.2006.01.008 16469402

[B15] Koonin EV Virology: Gulliver among the Lilliputians Curr Biol 2005 15 5 R167 169 10.1016/j.cub.2005.02.042 15753027

[B16] Filee J Pouget N Chandler M Phylogenetic evidence for extensive lateral acquisition of cellular genes by Nucleocytoplasmic large DNA viruses BMC Evol Biol 2008 8 320 10.1186/1471-2148-8-320 19036122 PMC2607284

[B17] Moreira D Brochier-Armanet C Giant viruses, giant chimeras: the multiple evolutionary histories of Mimivirus genes BMC Evol Biol 2008 8 12 10.1186/1471-2148-8-12 18205905 PMC2263039

[B18] Filee J Siguier P Chandler M I am what I eat and I eat what I am: acquisition of bacterial genes by giant viruses Trends Genet 2007 23 1 10 15 10.1016/j.tig.2006.11.002 17109990

[B19] Boyer M Yutin N Pagnier I Barrassi L Fournous G Espinosa M Robert C Azza A Sun S Rossmann MG Suzan-Monti M La Scola B Koonin EV Raoult D Giant Marseillevirus highlights the role of amoebae as a melting pot in emergence of chimaeric microorganisms Proc Natl Acad Sci USA 2009 in press 10.1073/pnas.0911354106 PMC2799887 20007369

[B20] Ghedin E Claverie JM Mimivirus relatives in the Sargasso sea Virol J 2005 2 62 10.1186/1743-422X-2-62 16105173 PMC1215527

[B21] Monier A Claverie JM Ogata H Taxonomic distribution of large DNA viruses in the sea Genome Biol 2008 9 7 R106 10.1186/gb-2008-9-7-r106 18598358 PMC2530865

[B22] Monier A Larsen JB Sandaa RA Bratbak G Claverie JM Ogata H Marine mimivirus relatives are probably large algal viruses Virol J 2008 5 12 10.1186/1743-422X-5-12 18215256 PMC2245910

[B23] Kristensen DM Mushegian AR Dolja VV Koonin EV New dimensions of the virus world discovered through metagenomics Trends Microbiol 2010 in press 10.1016/j.tim.2009.11.003 PMC3293453 19942437

[B24] Claverie JM Grzela R Lartigue A Bernadac A Nitsche S Vacelet J Ogata H Abergel C Mimivirus and Mimiviridae: giant viruses with an increasing number of potential hosts, including corals and sponges J Invertebr Pathol 2009 101 3 172 180 10.1016/j.jip.2009.03.011 19457438

[B25] Fitch WM Distinguishing homologous from analogous proteins Systematic Zoology 1970 19 99 106 10.2307/2412448 5449325

[B26] Koonin EV Orthologs, Paralogs and Evolutionary Genomics Annu Rev Genet 2005 39 309 338 16285863 10.1146/annurev.genet.39.073003.114725

[B27] Tatusov RL Fedorova ND Jackson JD Jacobs AR Kiryutin B Koonin EV Krylov DM Mazumder R Mekhedov SL Nikolskaya AN The COG database: an updated version includes eukaryotes BMC Bioinformatics 2003 4 41 10.1186/1471-2105-4-41 12969510 PMC222959

[B28] Tatusov RL Koonin EV Lipman DJ A genomic perspective on protein families Science 1997 278 5338 631 637 10.1126/science.278.5338.631 9381173

[B29] Mulkidjanian AY Koonin EV Makarova KS Mekhedov SL Sorokin A Wolf YI Dufresne A Partensky F Burd H Kaznadzey D Haselkorn R Galperin MY The cyanobacterial genome core and the origin of photosynthesis Proc Natl Acad Sci USA 2006 103 35 13126 13131 10.1073/pnas.0605709103 16924101 PMC1551899

[B30] Makarova KS Sorokin AV Novichkov PS Wolf YI Koonin EV Clusters of orthologous genes for 41 archaeal genomes and implications for evolutionary genomics of archaea Biol Direct 2007 2 33 10.1186/1745-6150-2-33 18042280 PMC2222616

[B31] Koonin EV Comparative genomics, minimal gene-sets and the last universal common ancestor Nat Rev Microbiol 2003 1 2 127 136 10.1038/nrmicro751 15035042

[B32] Charlebois RL Doolittle WF Computing prokaryotic gene ubiquity: rescuing the core from extinction Genome Res 2004 14 12 2469 2477 10.1101/gr.3024704 15574825 PMC534671

[B33] Koonin EV Mushegian AR Bork P Non-orthologous gene displacement Trends Genet 1996 12 9 334 336 10.1016/0168-9525(96)20010-1 8855656

[B34] Shimodaira H An approximately unbiased test of phylogenetic tree selection Syst Biol 2002 51 3 492 508 10.1080/10635150290069913 12079646

[B35] Ogata H Toyoda K Tomaru Y Nakayama N Shirai Y Claverie JM Nagasaki K Remarkable sequence similarity between the dinoflagellate-infecting marine girus and the terrestrial pathogen African swine fever virus Virol J 2009 6 178 10.1186/1743-422X-6-178 19860921 PMC2777158

[B36] Wolf YI Rogozin IB Grishin NV Koonin EV Genome trees and the tree of life Trends Genet 2002 18 9 472 479 10.1016/S0168-9525(02)02744-0 12175808

[B37] Csuros M Miklos I Streamlining and large ancestral genomes in Archaea inferred with a phylogenetic birth-and-death model Mol Biol Evol 2009 26 9 2087 2095 10.1093/molbev/msp123 19570746 PMC2726834

[B38] Senkevich TG Koonin EV Bugert JJ Darai G Moss B The genome of molluscum contagiosum virus: analysis and comparison with other poxviruses Virology 1997 233 1 19 42 10.1006/viro.1997.8607 9201214

[B39] Yutin N Koonin EV Evolution of DNA ligases of Nucleo-Cytoplasmic Large DNA viruses of eukaryotes: a case of hidden complexity Biology Direct 2009 4 1 51 10.1186/1745-6150-4-51 20021668 PMC2806865

[B40] Banerji S Aurass P Flieger A The manifold phospholipases A of Legionella pneumophila - identification, export, regulation, and their link to bacterial virulence Int J Med Microbiol 2008 298 3-4 169 181 10.1016/j.ijmm.2007.11.004 18178130

[B41] Koonin EV A duplicated catalytic motif in a new superfamily of phosphohydrolases and phospholipid synthases that includes poxvirus envelope proteins Trends Biochem Sci 1996 21 7 242 243 8755242

[B42] Husain M Moss B Similarities in the induction of post-Golgi vesicles by the vaccinia virus F13L protein and phospholipase D J Virol 2002 76 15 7777 7789 10.1128/JVI.76.15.7777-7789.2002 12097590 PMC136368

[B43] Koonin EV Wolf YI Nagasaki K Dolja VV The Big Bang of picorna-like virus evolution antedates the radiation of eukaryotic supergroups Nat Rev Microbiol 2008 6 12 925 939 10.1038/nrmicro2030 18997823

[B44] Lukashin AV Borodovsky M GeneMark.hmm: new solutions for gene finding Nucleic Acids Res 1998 26 4 1107 1115 10.1093/nar/26.4.1107 9461475 PMC147337

[B45] Altschul SF Madden TL Schaffer AA Zhang J Zhang Z Miller W Lipman DJ Gapped BLAST and PSI-BLAST: a new generation of protein database search programs Nucleic Acids Res 1997 25 17 3389 3402 10.1093/nar/25.17.3389 9254694 PMC146917

[B46] Edgar RC MUSCLE: multiple sequence alignment with high accuracy and high throughput Nucleic Acids Res 2004 32 5 1792 1797 10.1093/nar/gkh340 15034147 PMC390337

[B47] Marchler-Bauer A Bryant SH CD-Search: protein domain annotations on the fly Nucleic Acids Res 2004 32 Web Server W327 331 10.1093/nar/gkh454 15215404 PMC441592

[B48] Jobb G von Haeseler A Strimmer K TREEFINDER: a powerful graphical analysis environment for molecular phylogenetics BMC Evol Biol 2004 4 18 10.1186/1471-2148-4-18 15222900 PMC459214

[B49] Whelan S Goldman N A general empirical model of protein evolution derived from multiple protein families using a maximum-likelihood approach Mol Biol Evol 2001 18 5 691 699 11319253 10.1093/oxfordjournals.molbev.a003851

[B50] Puigbo P Garcia-Vallve S McInerney JO TOPD/FMTS: a new software to compare phylogenetic trees Bioinformatics 2007 23 12 1556 1558 10.1093/bioinformatics/btm135 17459965

[B51] Puigbo P Wolf YI Koonin EV Search for a 'Tree of Life' in the thicket of the phylogenetic forest J Biol 2009 8 6 59 10.1186/jbiol159 19594957 PMC2737373

[B52] Felsenstein J Inferring phylogenies from protein sequences by parsimony, distance, and likelihood methods Methods Enzymol 1996 266 418 427 full_text 8743697 10.1016/s0076-6879(96)66026-1

[B53] Csuros M Miklos I A probabilistic model for gene content evolution with duplication, loss, and horizontal transfer Lecture Notes in Computer Science 2006 3909 206 220 full_text

