# Supplementary material for: Eukaryotic large nucleo-cytoplasmic DNA viruses: Clusters of orthologous genes and reconstruction of viral genome evolution
Source: Virol J. 2009 Dec 17;6:223. doi: 10.1186/1743-422X-6-223 (PMC2806869; doi:10.1186/1743-422X-6-223)
Supplement: Additional file 1 — Functional classification of the 177 NCVOGs represented in two or more NCLDV families. [file 1743-422X-6-223-S1.DOC]

Supplemental Table S1. Functional classification of the 177 NCVOGs represented in two or more NCLDV families

| *cluster name* | *functional category* | *# of viral families* | *Number of genomes present in a cluster* | | | | | | *cluster annotation* |
| --- | --- | --- | --- | --- | --- | --- | --- | --- | --- |
|  |  |  | Pox-viridae | Asfa-viridae | Phycodna-viridae | Mimi-viridae | Irido- and Ascoviridae | Marcei-llevirus |  |
| NCVOG0023 | DNA replication, recombination and repair | 6 | 20 | 1 | 10 | 2 | 11 | 1 | D5-like helicase-primase |
| NCVOG0037 | DNA replication, recombination and repair | 6 | 1 | 1 | 8 | 2 | 2 | 1 | DNA topoisomerase II |
| NCVOG0038 | DNA replication, recombination and repair | 6 | 20 | 1 | 10 | 2 | 11 | 1 | DNA polymerase elongation subunit family B |
| NCVOG0076 | DNA replication, recombination and repair | 6 | 20 | 1 | 9 | 2 | 5 | 1 | DNA or RNA helicases of superfamily II (COG1061) |
| NCVOG1353 | Nucleotide metabolism | 6 | 3 | 1 | 10 | 2 | 7 | 1 | ribonucleoside diphosphate reductase, alpha subunit |
| NCVOG0276 | Nucleotide metabolism | 6 | 13 | 1 | 10 | 2 | 2 | 1 | Ribonucleotide reductase small subunit; apparent eukaryotic origin |
| NCVOG0052 | Other metabolic functions | 6 | 20 | 1 | 9 | 2 | 11 | 1 | disulfide (thiol) oxidoreductase; Erv1 / Alr family (pfam04777) |
| NCVOG1117 | Transcription and RNA processing | 6 | 20 | 1 | 8 | 2 | 1 | 1 | mRNA capping enzyme large subunit |
| NCVOG0236 | Transcription and RNA processing | 6 | 20 | 1 | 1 | 2 | 4 | 1 | Nudix hydrolase (D10 ortholog) |
| NCVOG1164 | Transcription and RNA processing | 6 | 20 | 1 | 10 | 2 | 10 | 1 | A1L transcription factor/late transcription factor VLTF-2; pfam03295: Pox_TAA1; Poxvirus trans-activator protein A1 C-terminal |
| NCVOG0262 | Transcription and RNA processing | 6 | 20 | 1 | 10 | 2 | 11 | 1 | pfam04947, Poxvirus Late Transcription Factor VLTF3 like |
| NCVOG0271 | Transcription and RNA processing | 6 | 20 | 1 | 1 | 2 | 11 | 1 | DNA-directed RNA polymerase subunit beta |
| NCVOG0272 | Transcription and RNA processing | 6 | 18 | 1 | 8 | 2 | 9 | 1 | Transcription factor S-II (TFIIS)-domain-containing protein |
| NCVOG0274 | Transcription and RNA processing | 6 | 20 | 1 | 1 | 2 | 11 | 1 | DNA-directed RNA polymerase subunit alpha |
| NCVOG1361 | Uncharacterized | 6 | 2 | 1 | 1 | 2 | 4 | 1 | pfam10544, T5orf172 domain |
| NCVOG0022 | Virion structure and morphogenesis | 6 | 20 | 1 | 10 | 2 | 11 | 1 | NCLDV major capsid protein (pfam03340 for Poxviridae; pfam04451 for others) |
| NCVOG0249 | Virion structure and morphogenesis | 6 | 20 | 1 | 10 | 2 | 11 | 1 | A32-like packaging ATPase |
| NCVOG1060 | DNA replication, recombination and repair | 5 | 20 | 0 | 1 | 2 | 11 | 1 | FLAP-like endonuclease XPG (cd00128) |
| NCVOG0278 | DNA replication, recombination and repair | 5 | 20 | 0 | 9 | 2 | 4 | 1 | RuvC, Holliday junction resolvases (HJRs); cl00243. Extended Pox_A22, Poxvirus A22 family (pfam04848). Marseille virus protein lacks C-term conserved positions. |
| NCVOG1136 | Nucleotide metabolism | 5 | 1 | 0 | 1 | 2 | 3 | 1 | bifunctional dihydrofolate reductase-thymidylate synthase |
| NCVOG0319 | Nucleotide metabolism | 5 | 15 | 1 | 1 | 2 | 0 | 1 | Thymidine kinase |
| NCVOG0330 | Signal transduction regulation | 5 | 16 | 0 | 4 | 2 | 3 | 1 | RING-finger-containing E3 ubiquitin ligase (COG5432: RAD18) |
| NCVOG0261 | Transcription and RNA processing | 5 | 20 | 1 | 0 | 2 | 11 | 1 | Poxvirus early transcription factor (VETF), large subunit (pfam04441) |
| NCVOG0273 | Transcription and RNA processing | 5 | 0 | 1 | 1 | 2 | 10 | 1 | divergent DNA-directed RNA polymerase subunit 5 |
| NCVOG1120 | Virion structure and morphogenesis | 5 | 2 | 0 | 7 | 2 | 5 | 1 | membrane-type matrix metalloproteinase; metallopeptidase WLM; pfam00413, cd04278, cd04275. |
| NCVOG0001 | DNA replication, recombination and repair | 4 | 0 | 0 | 7 | 2 | 1 | 1 | AAA family ATPase - unique in NCLDV |
| NCVOG0004 | DNA replication, recombination and repair | 4 | 2 | 1 | 0 | 2 | 0 | 1 | AP (apurinic) endonuclease family 2 - bacterial |
| NCVOG0031 | DNA replication, recombination and repair | 4 | 0 | 1 | 0 | 2 | 11 | 1 | unclassified DEAD/SNF2-like helicases |
| NCVOG0034 | DNA replication, recombination and repair | 4 | 11 | 1 | 6 | 0 | 0 | 1 | ATP-dependent DNA ligase (pfam01068, PRK01109) |
| NCVOG0241 | DNA replication, recombination and repair | 4 | 0 | 0 | 10 | 2 | 8 | 1 | Proliferating cell nuclear antigen (pfam02747, cd00577) |
| NCVOG1192 | DNA replication, recombination and repair | 4 | 1 | 1 | 9 | 2 | 0 | 0 | YqaJ viral recombinase family: pfam09588: This protein family is found in many different bacterial species but is of viral origin |
| NCVOG0248 | DNA replication, recombination and repair | 4 | 0 | 0 | 1 | 2 | 5 | 1 | Pif1 helicase |
| NCVOG1354 | DNA replication, recombination and repair | 4 | 0 | 0 | 7 | 2 | 11 | 1 | Ribonuclease III (cl00258) |
| NCVOG1154 | Host-virus interactions | 4 | 0 | 0 | 3 | 2 | 2 | 1 | SWIB/MDM2 domain-containing protein (chromatin condensation) |
| NCVOG0046 | Miscellaneous | 4 | 2 | 0 | 2 | 2 | 0 | 1 | DnaJ domain (pfam00226) |
| NCVOG1068 | Nucleotide metabolism | 4 | 17 | 1 | 8 | 0 | 4 | 0 | dUTPase (cl00493) |
| NCVOG0320 | Nucleotide metabolism | 4 | 4 | 1 | 5 | 0 | 11 | 0 | pfam02223: Thymidylate kinase |
| NCVOG1049 | Other metabolic functions | 4 | 9 | 0 | 5 | 2 | 6 | 0 | short chain dehydrogenase: This family contains a wide variety of dehydrogenases (pfam00106). |
| NCVOG0040 | Other metabolic functions | 4 | 19 | 0 | 6 | 2 | 3 | 0 | cd00127, DSPc, Dual specificity phosphatases (DSP); Ser/Thr and Tyr protein phosphatases |
| NCVOG0050 | Other metabolic functions | 4 | 4 | 0 | 5 | 2 | 2 | 0 | Esterase lipase superfamily (cl09107, pfam07859, pfam00135, COG0400) |
| NCVOG1087 | Other metabolic functions | 4 | 0 | 0 | 2 | 2 | 7 | 1 | papain-like cysteine peptidase (Cathepsin B group) |
| NCVOG0245 | Other metabolic functions | 4 | 0 | 0 | 7 | 2 | 4 | 1 | Patatin phospholipase |
| NCVOG0317 | Other metabolic functions | 4 | 0 | 0 | 8 | 2 | 7 | 1 | Thioredoxin |
| NCVOG0333 | Other metabolic functions | 4 | 3 | 0 | 2 | 0 | 1 | 1 | Ubiquitin |
| NCVOG1127 | Transcription and RNA processing | 4 | 0 | 1 | 7 | 2 | 0 | 1 | transcription initiation factor IIB |
| NCVOG0010 | Uncharacterized | 4 | 2 | 0 | 1 | 2 | 6 | 0 | pfam02498: Bro-N; BRO family, N-terminal domain: This family includes the N-terminus of baculovirus BRO and ALI motif proteins. |
| NCVOG0158 | Uncharacterized | 4 | 0 | 0 | 10 | 2 | 8 | 1 | unknown protein, conserved in irido-, phycodna-, mimi-, mama-, and Marseille viruses |
| NCVOG1083 | Uncharacterized | 4 | 0 | 0 | 7 | 2 | 2 | 1 | hypothetical protein |
| NCVOG1423 | Uncharacterized | 4 | 0 | 0 | 10 | 2 | 11 | 1 | uncharacterized, C-terminal domain conserved in irido, asco, phycodna, mimi |
| NCVOG0211 | Virion structure and morphogenesis | 4 | 20 | 1 | 0 | 2 | 11 | 0 | myristylated IMV envelope protein (pfam02442: Lipid membrane protein of large eukaryotic DNA viruses) |
| NCVOG0024 | DNA replication, recombination and repair | 3 | 0 | 1 | 0 | 2 | 0 | 1 | Superfamily II helicase related to herpesvirus replicative helicase (origin-binding protein UL9), pfam03121 |
| NCVOG0035 | DNA replication, recombination and repair | 3 | 3 | 0 | 0 | 2 | 2 | 0 | NAD+ dependent DNA ligase (smart00532) |
| NCVOG0036 | DNA replication, recombination and repair | 3 | 20 | 0 | 1 | 2 | 0 | 0 | DNA topoisomerase I |
| NCVOG0062 | DNA replication, recombination and repair | 3 | 0 | 0 | 7 | 2 | 4 | 0 | GIY-YIG-like endonuclease (pfam01541) |
| NCVOG0072 | DNA replication, recombination and repair | 3 | 0 | 0 | 9 | 2 | 0 | 1 | HNH endonucleases (as pfam01844), found in Marseille, mimi-, mama- and Phycodnaviridae |
| NCVOG0267 | DNA replication, recombination and repair | 3 | 20 | 1 | 0 | 2 | 0 | 0 | RNA-helicase DExH-NPH-II |
| NCVOG1352 | DNA replication, recombination and repair | 3 | 0 | 0 | 7 | 2 | 0 | 1 | ribonuclease H - bacterial; found in mimi-, mama, Phycodnaviridae, and Marseille virus; pfam00075 |
| NCVOG0308 | DNA replication, recombination and repair | 3 | 0 | 0 | 0 | 2 | 5 | 1 | DNA repair exonuclease (COG0419,COG0420) |
| NCVOG0312 | DNA replication, recombination and repair | 3 | 0 | 0 | 0 | 2 | 5 | 1 | COG1196, Smc, Chromosome segregation ATPases |
| NCVOG0435 | DNA replication, recombination and repair | 3 | 0 | 0 | 1 | 2 | 0 | 1 | AlkB, Alkylated DNA repair protein unique in NCLDV |
| NCVOG1045 | DNA replication, recombination and repair | 3 | 0 | 0 | 0 | 2 | 2 | 1 | pfam03159, XRN 5'-3' exonuclease |
| NCVOG0009 | Host-virus interactions | 3 | 2 | 1 | 0 | 0 | 1 | 0 | pfam00653: BIR (Baculovirus Inhibitor of apoptosis protein Repeat) domain |
| NCVOG1059 | Host-virus interactions | 3 | 2 | 1 | 0 | 0 | 2 | 0 | Apoptosis regulator Bcl-2 homolog (pfam00452); similar to N-half of eukaryotic MCL1 (myeloid cell leukemia sequence 1) protein |
| NCVOG0012 | Host-virus interactions | 3 | 18 | 1 | 1 | 0 | 0 | 0 | C-type lectin (poxvirus EEV glycoprotein etc); smart00034, cd03594,cd03593, pfam00059, cd00037, pfam05966 |
| NCVOG1074 | Host-virus interactions | 3 | 0 | 0 | 0 | 2 | 10 | 1 | restriction-fold endonuclease; D...H...D conserved. |
| NCVOG0002 | Miscellaneous | 3 | 1 | 0 | 5 | 2 | 0 | 0 | ABC transporter; pfam00005 |
| NCVOG1356 | Miscellaneous | 3 | 0 | 0 | 3 | 0 | 11 | 1 | 2-cysteine adaptor domain (pfam08793). The virus-specific domain is found fused to OTU/A20-like peptidases and S/T protein kinases. |
| NCVOG1358 | Miscellaneous | 3 | 11 | 0 | 0 | 2 | 0 | 1 | pfam00651, BTB/POZ domain: The BTB (for BR-C, ttk and bab) or POZ (for Pox virus and Zinc finger) domain. In mama, mimivirus it is often followed by WD-repeat; multiple paralogs in mimi, mamaviruses . Found on N-termini of some mimi-, mama- and Chordopoxvirinae proteins; in Chordopoxvirinae it is associated with Kelch domain. |
| NCVOG1360 | Miscellaneous | 3 | 15 | 0 | 0 | 2 | 1 | 0 | KilA domain (pfam04383); always is present at N-terminal except for mimiviruses. Sometimes is followed by a RING-finger domain |
| NCVOG0017 | Other metabolic functions | 3 | 2 | 0 | 1 | 2 | 0 | 0 | COG5092, NMT1, N-myristoyl transferase (pfam01233, pfam02799) |
| NCVOG1064 | Other metabolic functions | 3 | 0 | 0 | 8 | 2 | 0 | 1 | Cytidine and deoxycytidylate deaminase family (cd01283, cd01286) |
| NCVOG0058 | Other metabolic functions | 3 | 0 | 0 | 6 | 2 | 0 | 1 | Flavin-containing amine oxidoreductase conserved in mama-, mimi, Marseille virus, and Chloroviridae |
| NCVOG0063 | Other metabolic functions | 3 | 1 | 0 | 6 | 2 | 0 | 0 | GCN5-related N-acetyltransferases (GNAT; cd04301, pfam00583); Melanoplus sanguinipes entomopoxvirus has probably gained it independently |
| NCVOG0225 | Other metabolic functions | 3 | 2 | 0 | 2 | 0 | 0 | 1 | class 3 Lipase (cd00519, pfam01764) |
| NCVOG1114 | Other metabolic functions | 3 | 0 | 0 | 7 | 0 | 1 | 1 | inactivated thioredoxin/glutaredoxin |
| NCVOG1115 | Other metabolic functions | 3 | 20 | 0 | 0 | 2 | 0 | 1 | uracil-DNA glycosylase |
| NCVOG0246 | Other metabolic functions | 3 | 0 | 1 | 1 | 2 | 0 | 0 | pfam02902, Ulp1 protease family, C-terminal catalytic domain |
| NCVOG1166 | Other metabolic functions | 3 | 11 | 0 | 7 | 2 | 0 | 0 | pfam03336: Pox_C4_C10; Poxvirus C4/C10 protein; smart00702: P4Hc, Prolyl 4-hydroxylase alpha subunit homologues |
| NCVOG1191 | Other metabolic functions | 3 | 1 | 0 | 1 | 0 | 1 | 0 | SAM dependent methyltransferase (pfam08241, cd02440) |
| NCVOG1337 | Other metabolic functions | 3 | 2 | 0 | 1 | 0 | 3 | 0 | ELO, GNS1/SUR4 family (pfam01151). Elongation of very long chain fatty acids; eukaryotic |
| NCVOG0318 | Other metabolic functions | 3 | 2 | 0 | 6 | 2 | 0 | 0 | Thioredoxin-like proteins of Phycodnaviridae; in q3_Ectsi13242599 it was upstream to cd01049 domain (Ribonucleotide Reductase, R2/beta subunit (RNRR2)) |
| NCVOG0965 | Other metabolic functions | 3 | 2 | 1 | 0 | 0 | 1 | 0 | protein phosphatase 1, regulatory (inhibitor) subunit 15A (pfam10488) |
| NCVOG0295 | Signal transduction regulation | 3 | 20 | 0 | 5 | 2 | 0 | 0 | F10-like kinase |
| NCVOG1088 | Transcription and RNA processing | 3 | 0 | 1 | 0 | 0 | 11 | 1 | RNA ligase (conserved in irido-, asfa- asco- and Marseille viruses) |
| NCVOG1165 | Transcription and RNA processing | 3 | 20 | 1 | 0 | 2 | 0 | 0 | Pox_polyA_pol; Poxvirus poly(A) polymerase catalytic subunit |
| NCVOG1368 | Transcription and RNA processing | 3 | 18 | 1 | 0 | 0 | 1 | 0 | RNA polymerase, subunit 10; pfam05864, Chordopox_RPO7, Chordopoxvirus DNA-directed RNA polymerase 7 kDa polypeptide (RPO7) |
| NCVOG0229 | Uncharacterized | 3 | 0 | 0 | 2 | 2 | 4 | 0 | uncharacterized repeats |
| NCVOG1111 | Uncharacterized | 3 | 0 | 0 | 7 | 0 | 5 | 1 | uncharacterized family of NCLDV proteins found in iridoviridae, phycodnaviridae, ascoviridae, Marseille virus |
| NCVOG1175 | Uncharacterized | 3 | 16 | 0 | 3 | 2 | 0 | 0 | putative resolvase; cd00338, Ser_Recombinase, Serine Recombinase family, catalytic domain; pfam04708:Pox_F16; Poxvirus F16 protein |
| NCVOG1424 | Uncharacterized | 3 | 3 | 0 | 0 | 2 | 1 | 0 | uncharacterized domain; found downstream KilA, BRO, and MSV199 domains. Also is found in some baculoviruses (gi 165969059, 18138388) |
| NCVOG0632 | Uncharacterized | 3 | 0 | 0 | 9 | 2 | 0 | 1 | conserved hypotetical protein |
| NCVOG1122 | Virion structure and morphogenesis | 3 | 20 | 0 | 0 | 2 | 9 | 0 | Myristylated protein; pfam03003, DUF230 |
| NCVOG0047 | DNA replication, recombination and repair | 2 | 0 | 0 | 2 | 2 | 0 | 0 | DnaQ-like exonuclease (cd06135) and Exonuc_X-T Exonuclease (pfam00929) |
| NCVOG0199 | DNA replication, recombination and repair | 2 | 0 | 0 | 6 | 2 | 0 | 0 | uncharacterized protein |
| NCVOG0201 | DNA replication, recombination and repair | 2 | 12 | 0 | 0 | 0 | 2 | 0 | eukaryotic initiation factor 2a (eIF2a) -like protein |
| NCVOG0232 | DNA replication, recombination and repair | 2 | 0 | 0 | 4 | 0 | 0 | 1 | D12 class N6 adenine-specific DNA methyltransferase (pfam02086) |
| NCVOG1351 | DNA replication, recombination and repair | 2 | 0 | 0 | 1 | 2 | 0 | 0 | replication factor C small subunit 2 (PRK12402); cd00009, AAA, The AAA+ (ATPases Associated with a wide variety of cellular Activities) superfamily; pfam10424, RFC-E_C, Clamp-loader complex subunit E C-terminus |
| NCVOG0313 | DNA replication, recombination and repair | 2 | 0 | 0 | 7 | 2 | 0 | 0 | cl08263, TBP_TLF, TATA box binding protein (TBP) |
| NCVOG0335 | DNA replication, recombination and repair | 2 | 0 | 0 | 1 | 2 | 0 | 0 | UvrD/REP helicase (pfam00580) |
| NCVOG1075 | Host-virus interactions | 2 | 0 | 0 | 0 | 0 | 1 | 1 | restriction-fold endonuclease; E...D...EvK conserved |
| NCVOG0202 | Host-virus interactions | 2 | 14 | 0 | 0 | 0 | 1 | 0 | Immunoglobulin domain containing proteins (conserved YxC) |
| NCVOG1149 | Host-virus interactions | 2 | 10 | 0 | 0 | 0 | 1 | 0 | G protein-coupled receptor-like protein |
| NCVOG0309 | Host-virus interactions | 2 | 5 | 0 | 0 | 0 | 1 | 0 | Sema domain (pfam01403, cl08322) |
| NCVOG0071 | Miscellaneous | 2 | 0 | 0 | 1 | 0 | 5 | 0 | High Mobility Group (HMG)-box superfamily of DNA-binding proteins (cd01390) |
| NCVOG0349 | Miscellaneous | 2 | 0 | 0 | 0 | 2 | 0 | 1 | Zn-finger containing protein; similar to EGF-like domain containing protein |
| NCVOG1446 | Miscellaneous | 2 | 0 | 0 | 2 | 2 | 0 | 0 | cl00057, vWFA, Von Willebrand factor type A (vWA) domain |
| NCVOG1044 | Miscellaneous | 2 | 0 | 0 | 0 | 2 | 0 | 1 | WD-repeat family proteins (pfam00400) |
| NCVOG1046 | Miscellaneous | 2 | 0 | 0 | 4 | 2 | 0 | 0 | Zn-finger - containing protein |
| NCVOG1062 | Mobile elements | 2 | 1 | 0 | 0 | 0 | 1 | 0 | RNA-dependent DNA polymerase (cd01650) |
| NCVOG1067 | Nucleotide metabolism | 2 | 0 | 0 | 0 | 2 | 0 | 1 | deoxynucleotide monophosphate kinase (dNMP) kinase; Marseille virus, mama-, mimivirus only. |
| NCVOG0003 | Other metabolic functions | 2 | 0 | 0 | 1 | 2 | 0 | 0 | ADP-ribosylglycohydrolase (cl00614, pfam03747) |
| NCVOG1061 | Other metabolic functions | 2 | 2 | 0 | 0 | 2 | 0 | 0 | cd00143, PP2Cc, Serine/threonine phosphatases, family 2C |
| NCVOG0059 | Other metabolic functions | 2 | 0 | 1 | 0 | 2 | 0 | 0 | FtsJ-like methyltransferase family proteins (pfam01728) |
| NCVOG0061 | Other metabolic functions | 2 | 0 | 0 | 6 | 2 | 0 | 0 | asnB, asparagine synthetase B; mama-, mimi-, Phycodnavirus (PRK09431) |
| NCVOG0067 | Other metabolic functions | 2 | 0 | 0 | 9 | 2 | 0 | 0 | pfam00535: Glycosyl transferase family 2; COG1216: Predicted glycosyltransferases |
| NCVOG0070 | Other metabolic functions | 2 | 0 | 0 | 1 | 2 | 0 | 0 | Vacuolar (H+)-ATPase G subunit (pfam03179) |
| NCVOG0077 | Other metabolic functions | 2 | 1 | 0 | 2 | 0 | 0 | 0 | pfam02518: HATPase_c; Histidine kinase-, DNA gyrase B-, and HSP90-like ATPase: This family represents the structurally related ATPase domains of histidine kinase, DNA gyrase B and HSP90. |
| NCVOG1098 | Other metabolic functions | 2 | 0 | 0 | 1 | 0 | 11 | 0 | cl02680, NIF, NLI interacting factor-like phosphatase |
| NCVOG1128 | Other metabolic functions | 2 | 0 | 0 | 0 | 2 | 0 | 1 | glycosyl transferase |
| NCVOG0235 | Other metabolic functions | 2 | 0 | 0 | 2 | 2 | 0 | 0 | Nucleotidyl transferase: This family includes a wide range of enzymes which transfer nucleotides onto phosphosugars. (pfam00483; pfam02348: Cytidylyltransferase) |
| NCVOG1155 | Other metabolic functions | 2 | 0 | 0 | 2 | 0 | 3 | 0 | OTU-like cysteine protease |
| NCVOG0256 | Other metabolic functions | 2 | 20 | 0 | 0 | 2 | 0 | 0 | IMV envelope protein p35 |
| NCVOG1326 | Other metabolic functions | 2 | 10 | 0 | 5 | 0 | 0 | 0 | superoxide dismutase (cd00305, Cu-Zn_Superoxide_Dismutase) |
| NCVOG0310 | Other metabolic functions | 2 | 14 | 0 | 0 | 2 | 0 | 0 | Serpin (serine protease inhibitor); pfam00079, cd00172 |
| NCVOG0279 | Other metabolic functions | 2 | 0 | 0 | 8 | 2 | 0 | 0 | Putative methyl transferase (smart00317) |
| NCVOG0329 | Other metabolic functions | 2 | 0 | 1 | 0 | 2 | 0 | 0 | UBCc, Ubiquitin-conjugating enzyme E2 (cd00195) |
| NCVOG0331 | Other metabolic functions | 2 | 0 | 0 | 7 | 2 | 0 | 0 | Ubiquitin carboxyl-terminal hydrolase: pfam00443 |
| NCVOG0350 | Other metabolic functions | 2 | 0 | 0 | 0 | 2 | 0 | 1 | calcineurin-like phosphoesterase (pfam00149) |
| NCVOG0352 | Other metabolic functions | 2 | 0 | 0 | 0 | 2 | 0 | 1 | Metal dependent phosphohydrolase with conserved 'HD' motif |
| NCVOG0480 | Other metabolic functions | 2 | 0 | 0 | 0 | 2 | 0 | 1 | pfam01368, DHH, DHH family phosphohydrolase |
| NCVOG0970 | Other metabolic functions | 2 | 1 | 0 | 0 | 0 | 1 | 0 | cd02901, Macro_Poa1p_like, Macro domain, Poa1p_like family. One pox- and one iridovirus protein (possible independent HGT events) |
| NCVOG1004 | Other metabolic functions | 2 | 6 | 0 | 0 | 2 | 0 | 0 | photolyase |
| NCVOG1082 | Signal transduction regulation | 2 | 0 | 0 | 0 | 0 | 4 | 1 | divergent Serine/Threonine protein kinase |
| NCVOG0284 | Signal transduction regulation | 2 | 16 | 0 | 0 | 0 | 1 | 0 | ST kinase found in poxviruses and Iridovirus gi|15079100 |
| NCVOG0305 | Signal transduction regulation | 2 | 0 | 0 | 0 | 2 | 0 | 1 | Mimi-, mama-, Marseille virus tandem-duplicated ST (or Tyrosine) kinase; apparent eukaryotic origin (gi|66804679: protein kinase, TKL group [*Dictyostelium* *discoideum* AX4]) |
| NCVOG0075 | Transcription and RNA processing | 2 | 0 | 0 | 1 | 2 | 0 | 0 | Helix-turn-helix XRE-family like proteins (pfam01381, cd00093) |
| NCVOG1152 | Transcription and RNA processing | 2 | 20 | 0 | 0 | 1 | 0 | 0 | poly(A) polymerase small subunit PAPS |
| NCVOG0522 | Transcription and RNA processing | 2 | 0 | 1 | 0 | 2 | 0 | 0 | RNA polymerase subunit 6 |
| NCVOG0064 | Translation | 2 | 0 | 0 | 0 | 2 | 0 | 1 | GTP binding translation elongation factor |
| NCVOG0314 | Translation | 2 | 0 | 0 | 0 | 2 | 0 | 1 | Translation initiation factor SUI1 |
| NCVOG0979 | Translation | 2 | 0 | 0 | 0 | 2 | 0 | 1 | Eukaryotic peptide chain release (translation termination) factor 1 |
| NCVOG1051 | Uncharacterized | 2 | 0 | 0 | 0 | 2 | 3 | 0 | hypothetical protein conserved in ascovirus, mimi-, mamavirus |
| NCVOG0021 | Uncharacterized | 2 | 0 | 0 | 0 | 2 | 4 | 0 | collagen triple helix repeat containing protein (pfam01391) |
| NCVOG0090 | Uncharacterized | 2 | 1 | 0 | 0 | 0 | 1 | 0 | hypothetical protein with Zn fingers, found in *Amsacta* *moorei* entomopoxvirus and Invertebrate iridescent virus 6 |
| NCVOG1081 | Uncharacterized | 2 | 0 | 0 | 0 | 0 | 7 | 1 | hypothetical protein conserved in iridoviridae and Marseille virus |
| NCVOG1084 | Uncharacterized | 2 | 0 | 0 | 4 | 0 | 1 | 0 | hypothetical protein |
| NCVOG1106 | Uncharacterized | 2 | 0 | 0 | 1 | 0 | 5 | 0 | hypothetical Iridovirus and *Emiliania* *huxleyi* virus protein; tandem duplication in q2_Emihu73852638 |
| NCVOG0230 | Uncharacterized | 2 | 2 | 0 | 0 | 0 | 2 | 0 | MSV199 domain (pfam10553) |
| NCVOG1125 | Uncharacterized | 2 | 0 | 0 | 3 | 2 | 0 | 0 | protein of unknown function DUF305 conserved in bacteria; found in mimi-, mama-, Chloroviruses |
| NCVOG1129 | Uncharacterized | 2 | 0 | 0 | 7 | 2 | 0 | 0 | hypotetical mimi, mamavirus, Phycodnaviridae protein |
| NCVOG1130 | Uncharacterized | 2 | 0 | 0 | 1 | 2 | 0 | 0 | membrane protein with eukaryotic homologs (pfam03798: TRAM_LAG1_CLN8, TLC domain) |
| NCVOG1131 | Uncharacterized | 2 | 0 | 0 | 7 | 2 | 0 | 0 | hypotetical mimi, mamavirus, Phycodnaviridae protein |
| NCVOG1137 | Uncharacterized | 2 | 0 | 0 | 9 | 2 | 0 | 0 | hypothetical protein |
| NCVOG1216 | Uncharacterized | 2 | 0 | 0 | 7 | 2 | 0 | 0 | conserved hypotetical protein |
| NCVOG1271 | Uncharacterized | 2 | 0 | 0 | 6 | 0 | 0 | 1 | membrane-bound metal-dependent hydrolase; possible bacterial origin |
| NCVOG1342 | Uncharacterized | 2 | 0 | 0 | 8 | 2 | 0 | 0 | hypothetical mimi-, mama-, Phycodnavirus protein |
| NCVOG0315 | Uncharacterized | 2 | 6 | 0 | 0 | 0 | 2 | 0 | TNF-alpha-receptor-like protein (cd00185) |
| NCVOG1417 | Uncharacterized | 2 | 1 | 0 | 0 | 0 | 3 | 0 | hypothetical protein conserved in eukaryotes and other viruses |
| NCVOG0351 | Uncharacterized | 2 | 0 | 0 | 0 | 2 | 0 | 1 | cell division cycle 123 homolog |
| NCVOG0423 | Uncharacterized | 2 | 2 | 0 | 0 | 0 | 1 | 0 | vascular endothelial growth factor-like protein (cd00135) |
| NCVOG0436 | Uncharacterized | 2 | 0 | 0 | 0 | 2 | 0 | 1 | short hypothetical protein |
| NCVOG0507 | Uncharacterized | 2 | 0 | 0 | 0 | 2 | 0 | 1 | hypothetical protein of Marseille virus, mama-, mimivirus. |
| NCVOG0518 | Uncharacterized | 2 | 0 | 1 | 0 | 2 | 0 | 0 | hypothetical protein |
| NCVOG0575 | Uncharacterized | 2 | 0 | 1 | 0 | 2 | 0 | 0 | hypothetical protein |
| NCVOG0645 | Uncharacterized | 2 | 0 | 0 | 6 | 2 | 0 | 0 | hypotetical protein of Chlorovirus and Mimiviruses |
| NCVOG0716 | Uncharacterized | 2 | 0 | 0 | 0 | 2 | 0 | 1 | P-loop ATPase or GTPase |
| NCVOG0772 | Uncharacterized | 2 | 0 | 0 | 0 | 2 | 0 | 1 | Uncharacterized protein conserved in bacteria, Marseille virus, mama- and mimivirus |
| NCVOG0773 | Uncharacterized | 2 | 0 | 0 | 0 | 2 | 2 | 0 | hypotetical protein of mama-, mimiviruses |
| NCVOG0815 | Uncharacterized | 2 | 0 | 0 | 0 | 2 | 0 | 1 | Uncharacterized protein conserved in bacteria, Marseille virus, mama- and mimivirus |
| NCVOG0966 | Uncharacterized | 2 | 0 | 0 | 0 | 0 | 2 | 1 | hypothetical protein |
| NCVOG0968 | Uncharacterized | 2 | 0 | 0 | 0 | 0 | 2 | 1 | hypothetical protein |
| NCVOG0971 | Uncharacterized | 2 | 0 | 0 | 0 | 0 | 1 | 1 | hypothetical protein |
| NCVOG0978 | Uncharacterized | 2 | 2 | 0 | 0 | 0 | 1 | 0 | SORF2 protein of herpesvirus; pfam02393: US22 like: This is the US22 protein family of hypothetical proteins from herpes virus. |
| NCVOG0980 | Uncharacterized | 2 | 0 | 0 | 1 | 0 | 0 | 1 | hypothetical protein PBCV1_A579L |
| NCVOG0981 | Uncharacterized | 2 | 0 | 0 | 1 | 0 | 0 | 1 | hypothetical membrane protein |
| NCVOG0982 | Uncharacterized | 2 | 0 | 0 | 1 | 0 | 0 | 1 | putative peptidoglycan peptidase |
| NCVOG1162 | Virion structure and morphogenesis | 2 | 20 | 0 | 0 | 2 | 0 | 0 | Poxvirus P4B major core protein; conserved in Poxviridae, mama, mimivirus; pfam03292: Pox_P4B |
