# Supplementary material for: Eukaryotic large nucleo-cytoplasmic DNA viruses: Clusters of orthologous genes and reconstruction of viral genome evolution
Source: Virol J. 2009 Dec 17;6:223. doi: 10.1186/1743-422X-6-223 (PMC2806869; doi:10.1186/1743-422X-6-223)
Supplement: Additional file 2 — The ML trees for 10 (nearly) universal NCLDV proteins: D5-like helicase-primase (D5_helicase_primase); Family B DNA polymerase (DNApol_B); A32-like packaging ATPase (Pox_A32_pfam04665); A2L-like transcription factor (Pox_VLTF3); Ribonucleotide reductase, small subunit; RNA polymerase, α-subunit; RNA polymerase, β-subunit;superfamily II helicase; mRNA capping enzyme, large subunit; Myristylated envelope protein. [file 1743-422X-6-223-S2.PPT]

## Slide 1
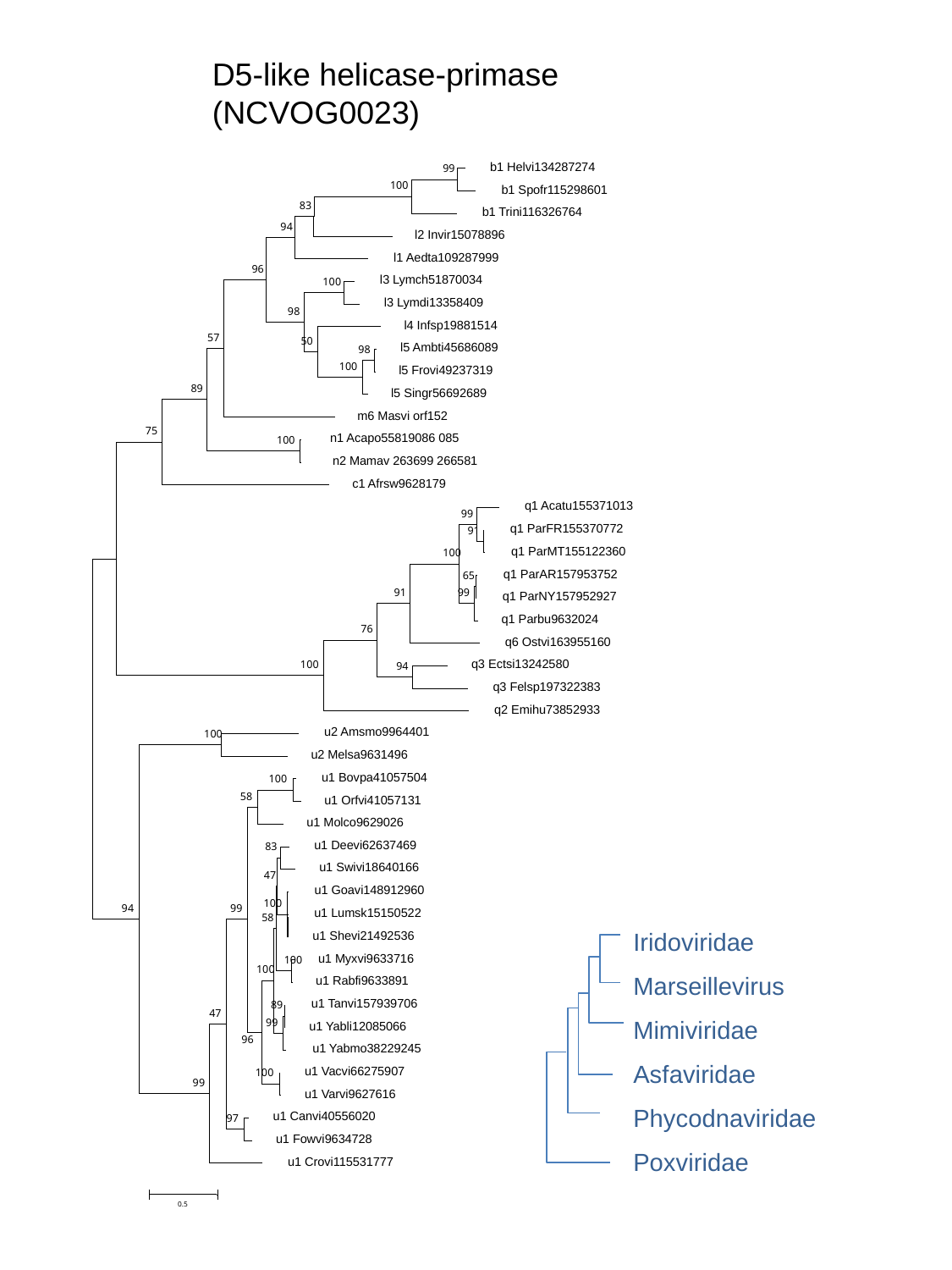

D5-like helicase-primase
(NCVOG0023)
 b1 Helvi134287274
99
100
 b1 Spofr115298601
83
 b1 Trini116326764
94
 l2 Invir15078896
 l1 Aedta109287999
96
 l3 Lymch51870034
100
 l3 Lymdi13358409
98
 l4 Infsp19881514
57
50
 l5 Ambti45686089
98
100
 l5 Frovi49237319
89
 l5 Singr56692689
 m6 Masvi orf152
75
 n1 Acapo55819086 085
100
 n2 Mamav 263699 266581
 c1 Afrsw9628179
 q1 Acatu155371013
99
 q1 ParFR155370772
91
 q1 ParMT155122360
100
 q1 ParAR157953752
65
91
99
 q1 ParNY157952927
 q1 Parbu9632024
76
 q6 Ostvi163955160
 q3 Ectsi13242580
100
94
 q3 Felsp197322383
 q2 Emihu73852933
 u2 Amsmo9964401
100
 u2 Melsa9631496
 u1 Bovpa41057504
100
58
 u1 Orfvi41057131
 u1 Molco9629026
 u1 Deevi62637469
83
 u1 Swivi18640166
47
 u1 Goavi148912960
100
94
99
 u1 Lumsk15150522
58
 u1 Shevi21492536
 u1 Myxvi9633716
100
100
 u1 Rabfi9633891
 u1 Tanvi157939706
89
47
99
 u1 Yabli12085066
96
 u1 Yabmo38229245
 u1 Vacvi66275907
100
99
 u1 Varvi9627616
 u1 Canvi40556020
97
 u1 Fowvi9634728
 u1 Crovi115531777
0.5
Iridoviridae
Marseillevirus
Mimiviridae
Asfaviridae
Phycodnaviridae
Poxviridae

## Slide 2
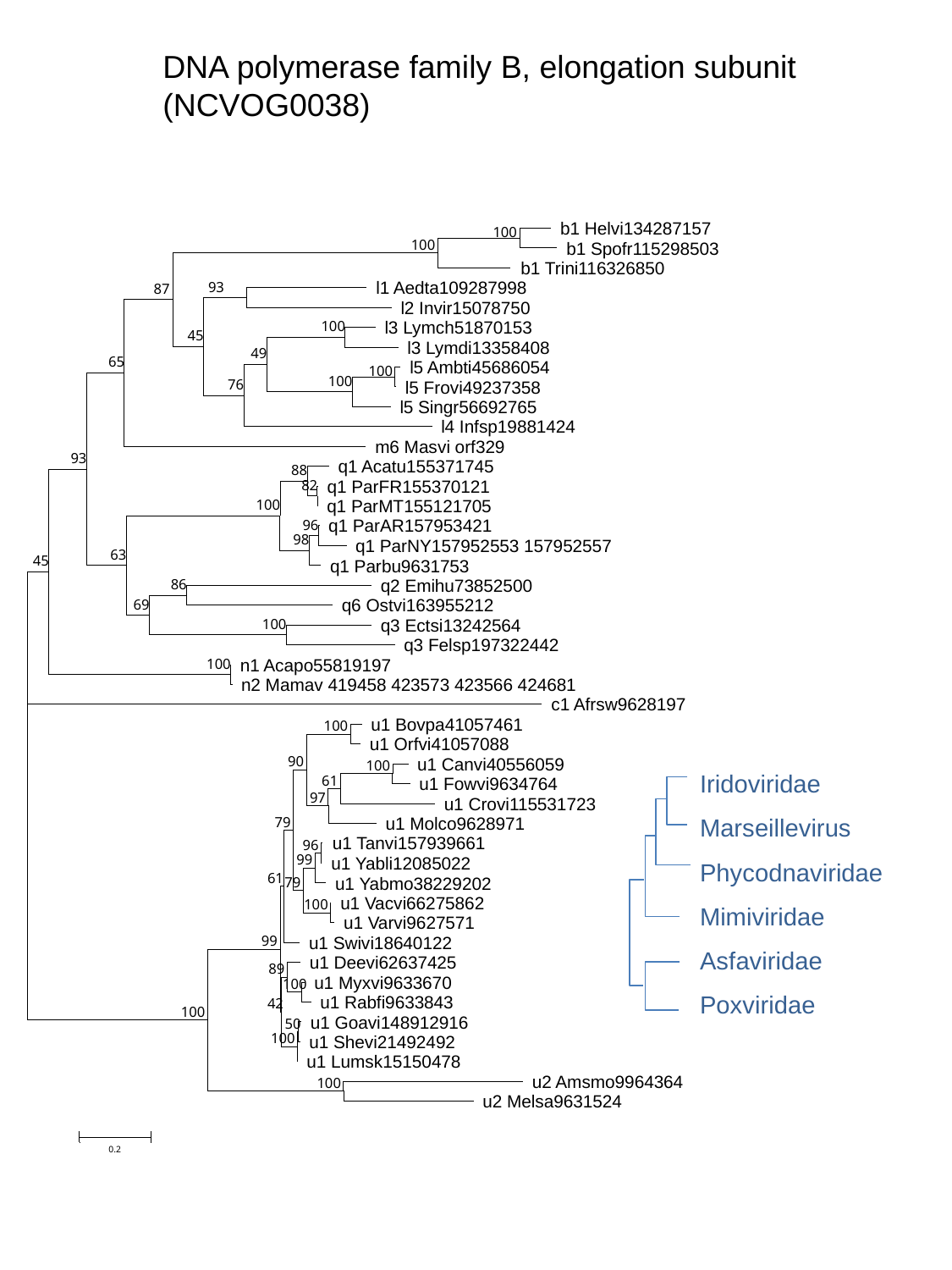

DNA polymerase family B, elongation subunit
(NCVOG0038)
 b1 Helvi134287157
100
100
 b1 Spofr115298503
 b1 Trini116326850
 l1 Aedta109287998
93
87
 l2 Invir15078750
 l3 Lymch51870153
100
45
 l3 Lymdi13358408
49
65
 l5 Ambti45686054
100
100
76
 l5 Frovi49237358
 l5 Singr56692765
 l4 Infsp19881424
 m6 Masvi orf329
93
 q1 Acatu155371745
88
 q1 ParFR155370121
82
 q1 ParMT155121705
100
 q1 ParAR157953421
96
98
 q1 ParNY157952553 157952557
63
45
 q1 Parbu9631753
 q2 Emihu73852500
86
 q6 Ostvi163955212
69
 q3 Ectsi13242564
100
 q3 Felsp197322442
 n1 Acapo55819197
100
 n2 Mamav 419458 423573 423566 424681
 c1 Afrsw9628197
 u1 Bovpa41057461
100
 u1 Orfvi41057088
90
 u1 Canvi40556059
100
61
 u1 Fowvi9634764
97
 u1 Crovi115531723
 u1 Molco9628971
79
 u1 Tanvi157939661
96
99
 u1 Yabli12085022
61
 u1 Yabmo38229202
79
 u1 Vacvi66275862
100
 u1 Varvi9627571
 u1 Swivi18640122
99
 u1 Deevi62637425
89
 u1 Myxvi9633670
100
 u1 Rabfi9633843
42
100
 u1 Goavi148912916
50
100
 u1 Shevi21492492
 u1 Lumsk15150478
 u2 Amsmo9964364
100
 u2 Melsa9631524
0.2
Iridoviridae
Marseillevirus
Phycodnaviridae
Mimiviridae
Asfaviridae
Poxviridae

## Slide 3
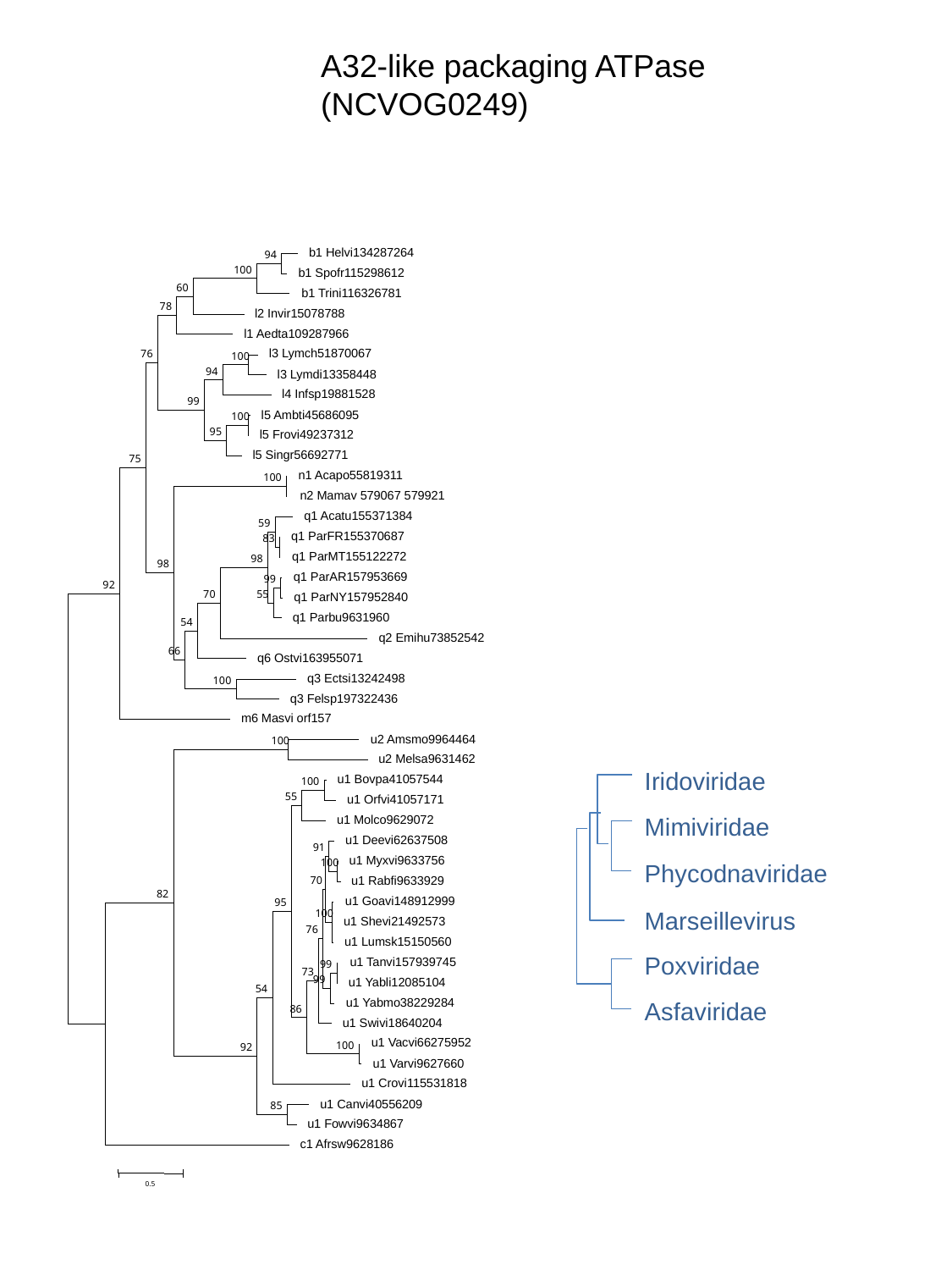

A32-like packaging ATPase
(NCVOG0249)
 b1 Helvi134287264
94
100
 b1 Spofr115298612
60
 b1 Trini116326781
78
 l2 Invir15078788
 l1 Aedta109287966
 l3 Lymch51870067
76
100
94
 l3 Lymdi13358448
 l4 Infsp19881528
99
 l5 Ambti45686095
100
95
 l5 Frovi49237312
 l5 Singr56692771
75
 n1 Acapo55819311
100
 n2 Mamav 579067 579921
 q1 Acatu155371384
59
 q1 ParFR155370687
83
 q1 ParMT155122272
98
98
 q1 ParAR157953669
99
92
70
55
 q1 ParNY157952840
 q1 Parbu9631960
54
 q2 Emihu73852542
66
 q6 Ostvi163955071
 q3 Ectsi13242498
100
 q3 Felsp197322436
 m6 Masvi orf157
 u2 Amsmo9964464
100
 u2 Melsa9631462
 u1 Bovpa41057544
100
55
 u1 Orfvi41057171
 u1 Molco9629072
 u1 Deevi62637508
91
 u1 Myxvi9633756
100
 u1 Rabfi9633929
70
82
 u1 Goavi148912999
95
100
 u1 Shevi21492573
76
 u1 Lumsk15150560
 u1 Tanvi157939745
99
73
99
 u1 Yabli12085104
54
 u1 Yabmo38229284
86
 u1 Swivi18640204
 u1 Vacvi66275952
100
92
 u1 Varvi9627660
 u1 Crovi115531818
 u1 Canvi40556209
85
 u1 Fowvi9634867
 c1 Afrsw9628186
0.5
Iridoviridae
Mimiviridae
Phycodnaviridae
Marseillevirus
Poxviridae
Asfaviridae

## Slide 4
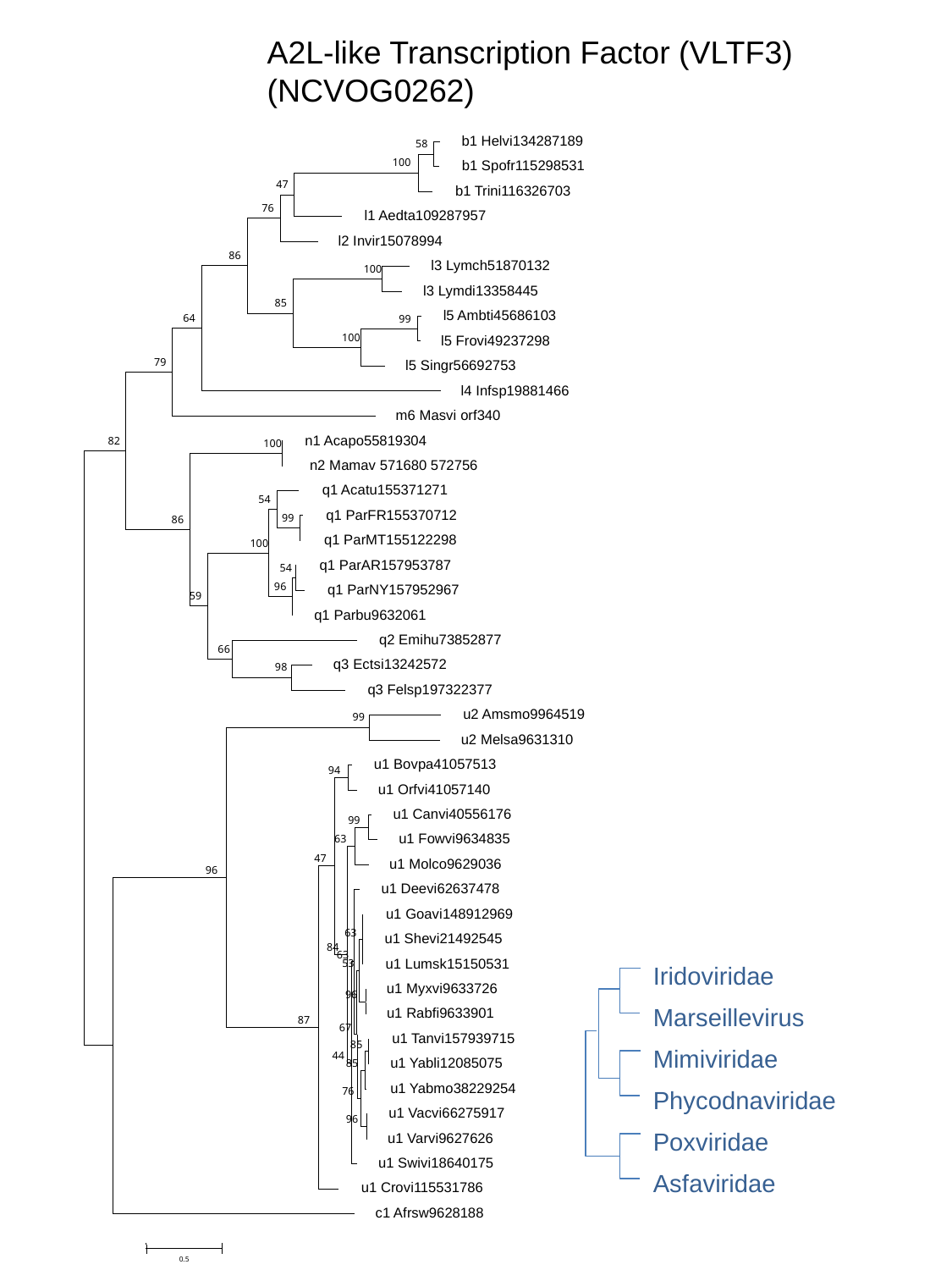

A2L-like Transcription Factor (VLTF3)
(NCVOG0262)
 b1 Helvi134287189
58
100
 b1 Spofr115298531
47
 b1 Trini116326703
76
 l1 Aedta109287957
 l2 Invir15078994
86
 l3 Lymch51870132
100
 l3 Lymdi13358445
85
 l5 Ambti45686103
64
99
100
 l5 Frovi49237298
79
 l5 Singr56692753
 l4 Infsp19881466
 m6 Masvi orf340
 n1 Acapo55819304
82
100
 n2 Mamav 571680 572756
 q1 Acatu155371271
54
 q1 ParFR155370712
99
86
 q1 ParMT155122298
100
 q1 ParAR157953787
54
96
 q1 ParNY157952967
59
 q1 Parbu9632061
 q2 Emihu73852877
66
 q3 Ectsi13242572
98
 q3 Felsp197322377
 u2 Amsmo9964519
99
 u2 Melsa9631310
 u1 Bovpa41057513
94
 u1 Orfvi41057140
 u1 Canvi40556176
99
 u1 Fowvi9634835
63
47
 u1 Molco9629036
96
 u1 Deevi62637478
 u1 Goavi148912969
63
 u1 Shevi21492545
84
63
 u1 Lumsk15150531
53
 u1 Myxvi9633726
96
 u1 Rabfi9633901
87
67
 u1 Tanvi157939715
85
44
 u1 Yabli12085075
85
 u1 Yabmo38229254
76
 u1 Vacvi66275917
96
 u1 Varvi9627626
 u1 Swivi18640175
 u1 Crovi115531786
 c1 Afrsw9628188
0.5
Iridoviridae
Marseillevirus
Mimiviridae
Phycodnaviridae
Poxviridae
Asfaviridae

## Slide 5
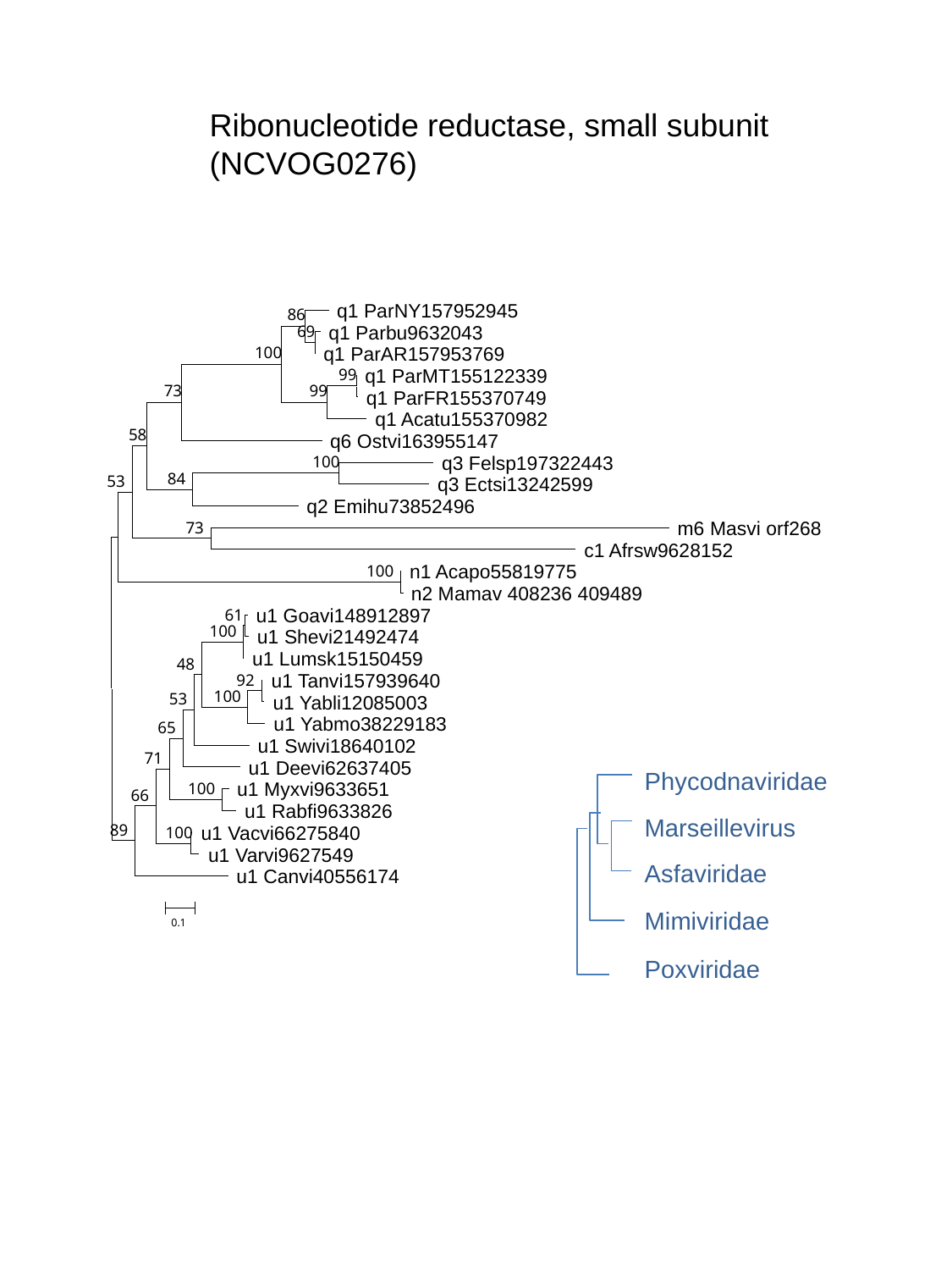

Ribonucleotide reductase, small subunit
(NCVOG0276)
 q1 ParNY157952945
86
 q1 Parbu9632043
69
 q1 ParAR157953769
100
 q1 ParMT155122339
99
73
99
 q1 ParFR155370749
 q1 Acatu155370982
58
 q6 Ostvi163955147
 q3 Felsp197322443
100
84
53
 q3 Ectsi13242599
 q2 Emihu73852496
 m6 Masvi orf268
73
 c1 Afrsw9628152
 n1 Acapo55819775
100
 n2 Mamav 408236 409489
 u1 Goavi148912897
61
100
 u1 Shevi21492474
 u1 Lumsk15150459
48
 u1 Tanvi157939640
92
100
53
 u1 Yabli12085003
 u1 Yabmo38229183
65
 u1 Swivi18640102
71
 u1 Deevi62637405
 u1 Myxvi9633651
100
66
 u1 Rabfi9633826
89
 u1 Vacvi66275840
100
 u1 Varvi9627549
 u1 Canvi40556174
0.1
Phycodnaviridae
Marseillevirus
Asfaviridae
Mimiviridae
Poxviridae

## Slide 6
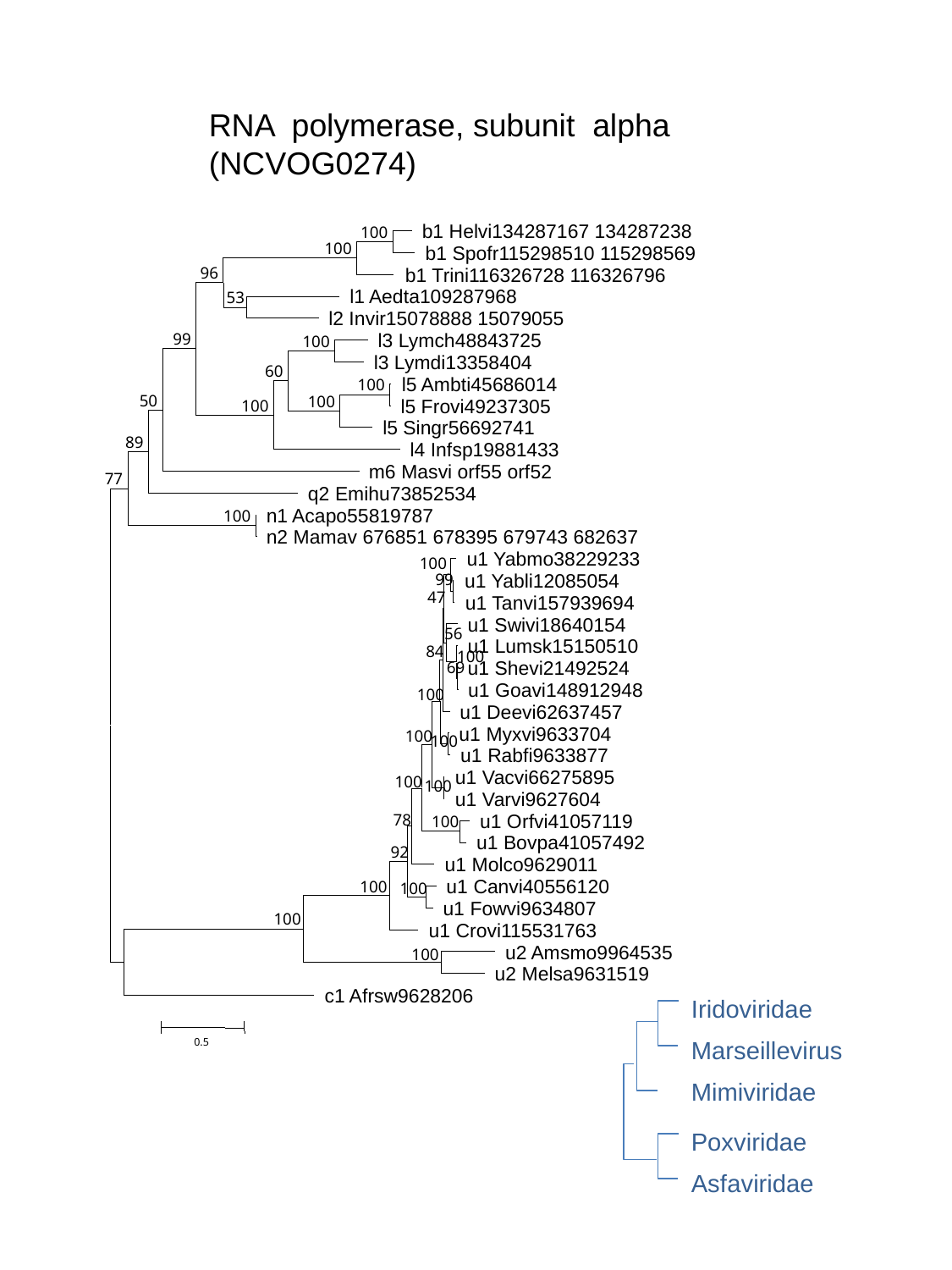

RNA polymerase, subunit alpha
(NCVOG0274)
 b1 Helvi134287167 134287238
100
100
 b1 Spofr115298510 115298569
 b1 Trini116326728 116326796
96
 l1 Aedta109287968
53
 l2 Invir15078888 15079055
 l3 Lymch48843725
99
100
 l3 Lymdi13358404
60
 l5 Ambti45686014
100
50
100
 l5 Frovi49237305
100
 l5 Singr56692741
89
 l4 Infsp19881433
 m6 Masvi orf55 orf52
77
 q2 Emihu73852534
 n1 Acapo55819787
100
 n2 Mamav 676851 678395 679743 682637
 u1 Yabmo38229233
100
 u1 Yabli12085054
99
47
 u1 Tanvi157939694
 u1 Swivi18640154
56
 u1 Lumsk15150510
84
100
 u1 Shevi21492524
69
 u1 Goavi148912948
100
 u1 Deevi62637457
 u1 Myxvi9633704
100
100
 u1 Rabfi9633877
 u1 Vacvi66275895
100
100
 u1 Varvi9627604
 u1 Orfvi41057119
78
100
 u1 Bovpa41057492
92
 u1 Molco9629011
 u1 Canvi40556120
100
100
 u1 Fowvi9634807
100
 u1 Crovi115531763
 u2 Amsmo9964535
100
 u2 Melsa9631519
 c1 Afrsw9628206
0.5
Iridoviridae
Marseillevirus
Mimiviridae
Poxviridae
Asfaviridae

## Slide 7
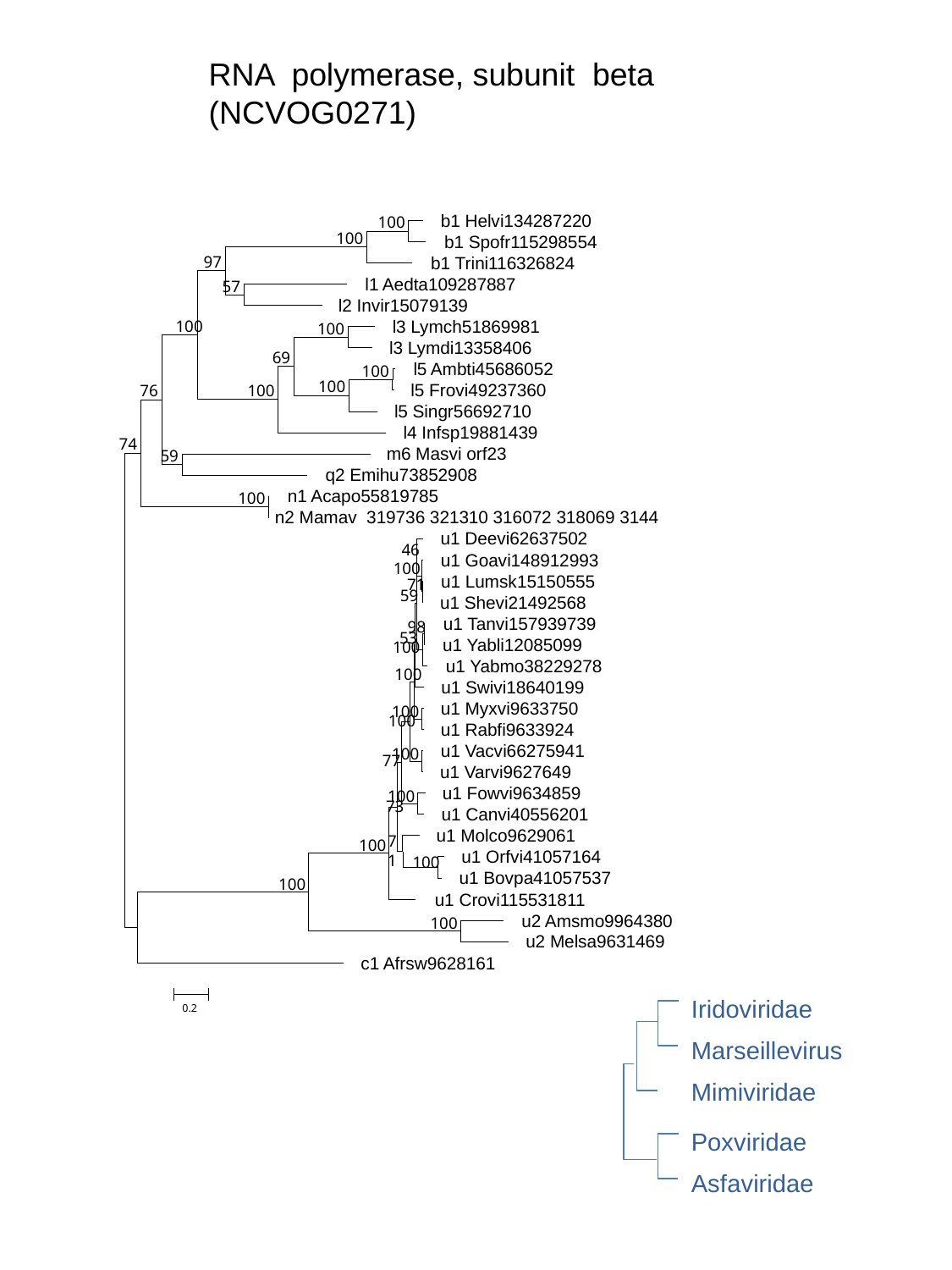

RNA polymerase, subunit beta
(NCVOG0271)
 b1 Helvi134287220
100
100
 b1 Spofr115298554
 b1 Trini116326824
97
 l1 Aedta109287887
57
 l2 Invir15079139
 l3 Lymch51869981
100
100
 l3 Lymdi13358406
69
 l5 Ambti45686052
100
100
 l5 Frovi49237360
100
76
 l5 Singr56692710
 l4 Infsp19881439
74
 m6 Masvi orf23
59
 q2 Emihu73852908
 n1 Acapo55819785
100
 n2 Mamav 319736 321310 316072 318069 3144
 u1 Deevi62637502
46
 u1 Goavi148912993
100
 u1 Lumsk15150555
71
59
 u1 Shevi21492568
 u1 Tanvi157939739
98
53
 u1 Yabli12085099
100
 u1 Yabmo38229278
100
 u1 Swivi18640199
 u1 Myxvi9633750
100
100
 u1 Rabfi9633924
 u1 Vacvi66275941
100
77
 u1 Varvi9627649
 u1 Fowvi9634859
100
73
 u1 Canvi40556201
 u1 Molco9629061
71
100
 u1 Orfvi41057164
100
 u1 Bovpa41057537
100
 u1 Crovi115531811
 u2 Amsmo9964380
100
 u2 Melsa9631469
 c1 Afrsw9628161
0.2
Iridoviridae
Marseillevirus
Mimiviridae
Poxviridae
Asfaviridae

## Slide 8
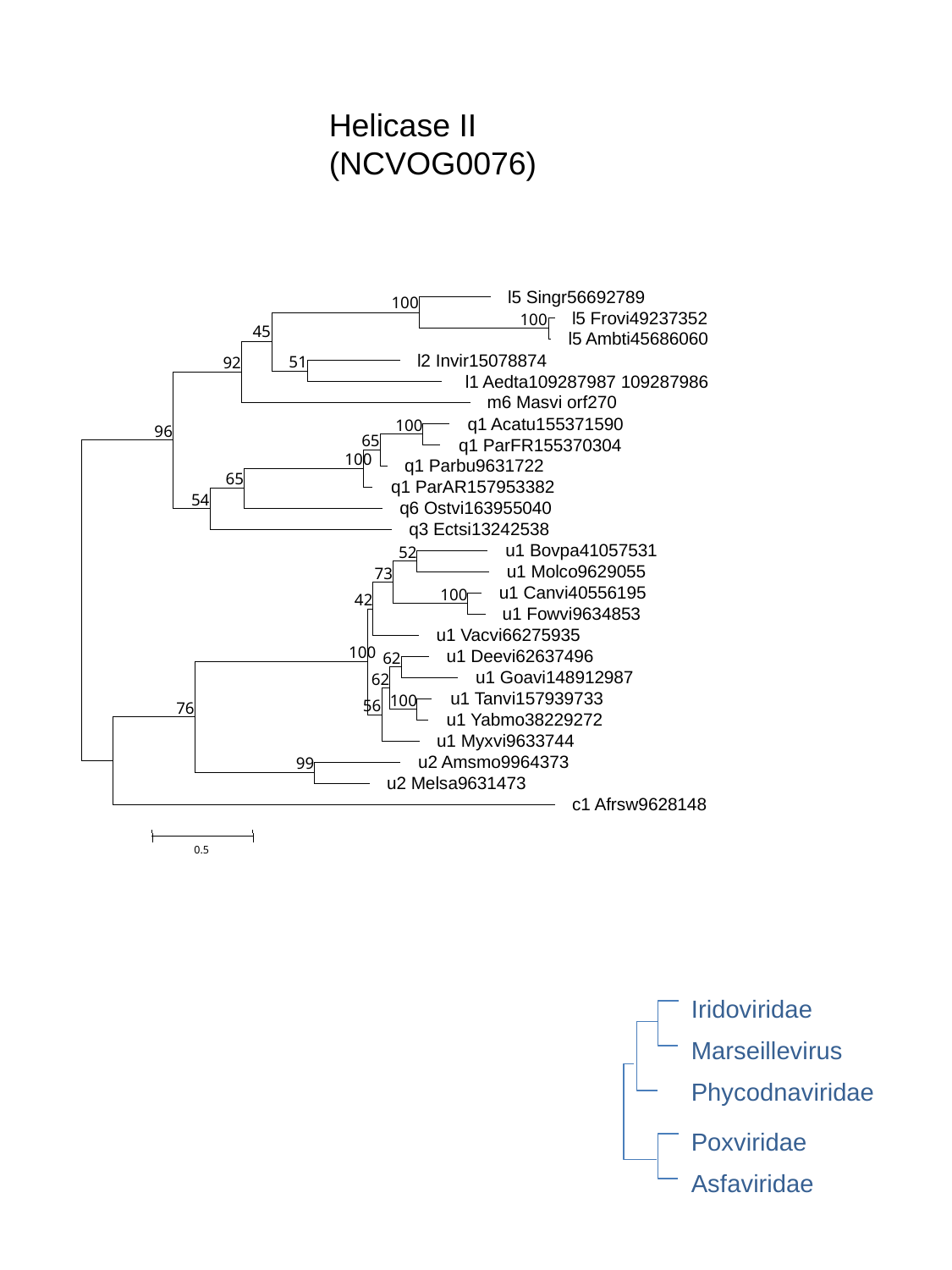

Helicase II
(NCVOG0076)
 l5 Singr56692789
100
 l5 Frovi49237352
100
45
 l5 Ambti45686060
 l2 Invir15078874
51
92
 l1 Aedta109287987 109287986
 m6 Masvi orf270
 q1 Acatu155371590
100
96
65
 q1 ParFR155370304
100
 q1 Parbu9631722
65
 q1 ParAR157953382
54
 q6 Ostvi163955040
 q3 Ectsi13242538
 u1 Bovpa41057531
52
 u1 Molco9629055
73
 u1 Canvi40556195
100
42
 u1 Fowvi9634853
 u1 Vacvi66275935
100
 u1 Deevi62637496
62
 u1 Goavi148912987
62
 u1 Tanvi157939733
100
56
76
 u1 Yabmo38229272
 u1 Myxvi9633744
 u2 Amsmo9964373
99
 u2 Melsa9631473
 c1 Afrsw9628148
0.5
Iridoviridae
Marseillevirus
Phycodnaviridae
Poxviridae
Asfaviridae

## Slide 9
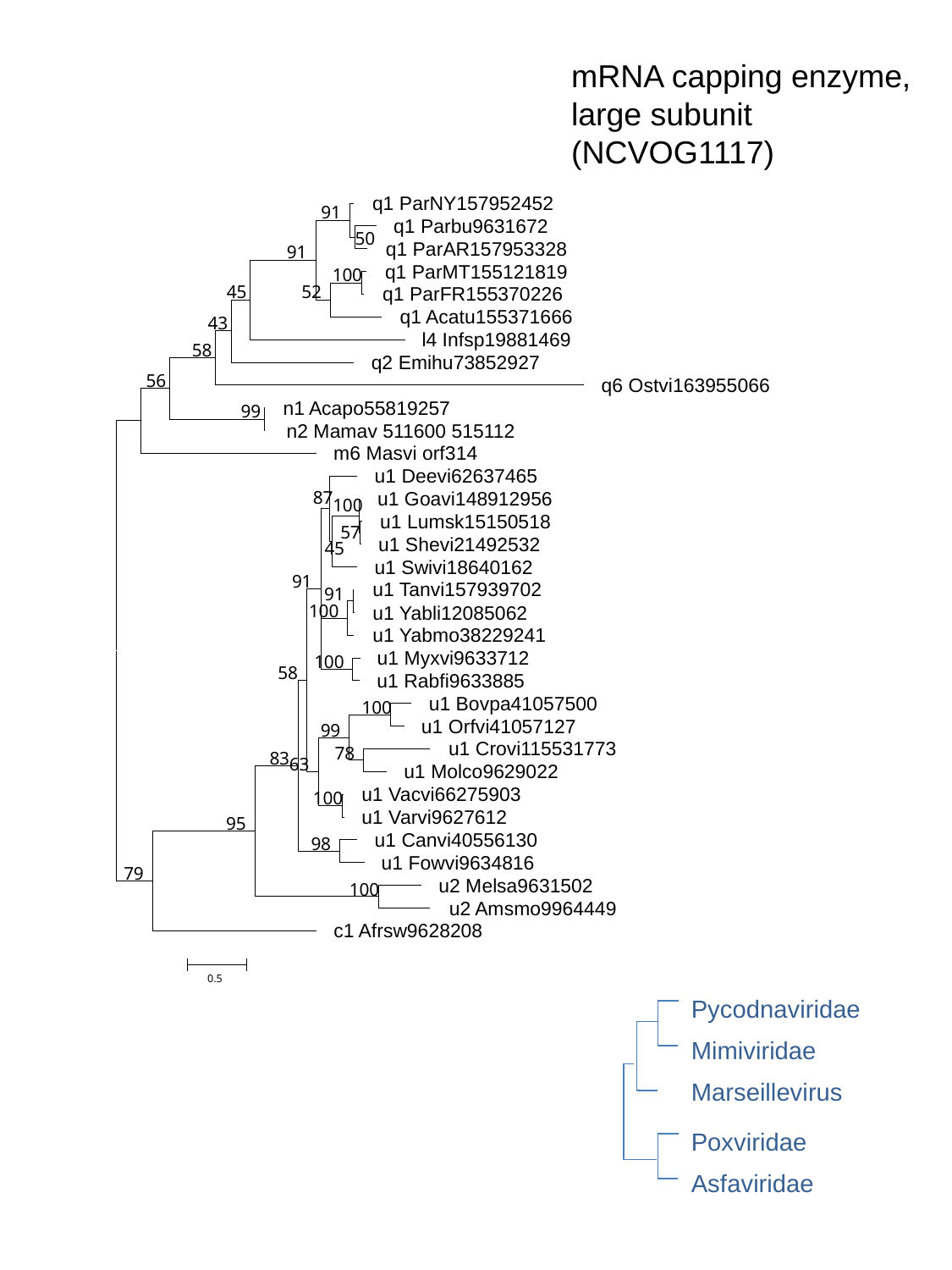

mRNA capping enzyme, large subunit (NCVOG1117)
 q1 ParNY157952452
91
 q1 Parbu9631672
50
 q1 ParAR157953328
91
 q1 ParMT155121819
100
45
52
 q1 ParFR155370226
 q1 Acatu155371666
43
 l4 Infsp19881469
58
 q2 Emihu73852927
56
 q6 Ostvi163955066
 n1 Acapo55819257
99
 n2 Mamav 511600 515112
 m6 Masvi orf314
 u1 Deevi62637465
87
 u1 Goavi148912956
100
 u1 Lumsk15150518
57
 u1 Shevi21492532
45
 u1 Swivi18640162
91
 u1 Tanvi157939702
91
100
 u1 Yabli12085062
 u1 Yabmo38229241
 u1 Myxvi9633712
100
58
 u1 Rabfi9633885
 u1 Bovpa41057500
100
 u1 Orfvi41057127
99
 u1 Crovi115531773
78
83
63
 u1 Molco9629022
 u1 Vacvi66275903
100
 u1 Varvi9627612
95
 u1 Canvi40556130
98
 u1 Fowvi9634816
79
 u2 Melsa9631502
100
 u2 Amsmo9964449
 c1 Afrsw9628208
0.5
Pycodnaviridae
Mimiviridae
Marseillevirus
Poxviridae
Asfaviridae

## Slide 10
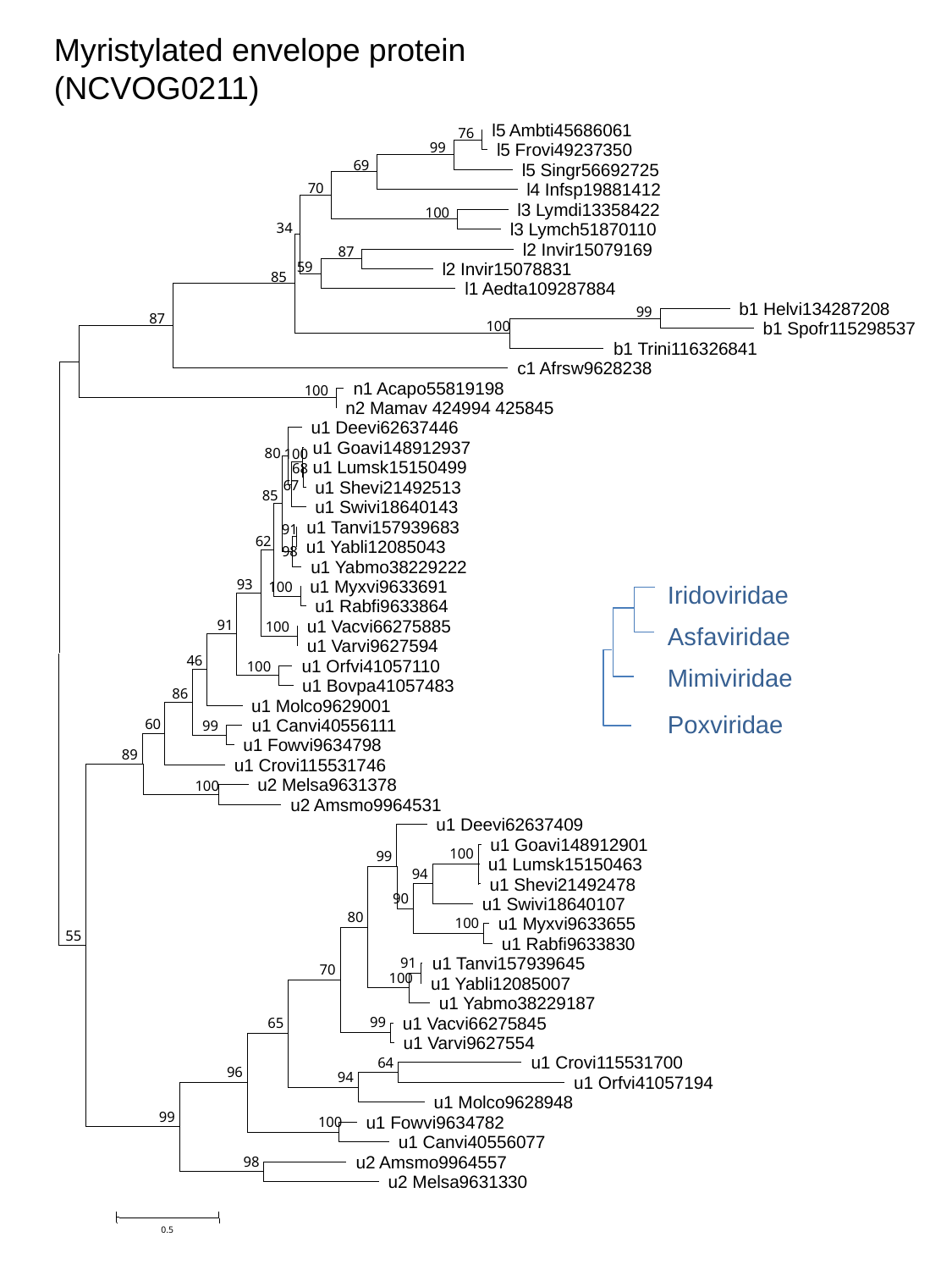

Myristylated envelope protein
(NCVOG0211)
 l5 Ambti45686061
76
99
 l5 Frovi49237350
69
 l5 Singr56692725
 l4 Infsp19881412
70
 l3 Lymdi13358422
100
 l3 Lymch51870110
34
 l2 Invir15079169
87
59
 l2 Invir15078831
85
 l1 Aedta109287884
 b1 Helvi134287208
99
87
100
 b1 Spofr115298537
 b1 Trini116326841
 c1 Afrsw9628238
 n1 Acapo55819198
100
 n2 Mamav 424994 425845
 u1 Deevi62637446
 u1 Goavi148912937
80
100
 u1 Lumsk15150499
68
 u1 Shevi21492513
67
85
 u1 Swivi18640143
 u1 Tanvi157939683
91
 u1 Yabli12085043
98
 u1 Yabmo38229222
 u1 Myxvi9633691
100
 u1 Rabfi9633864
 u1 Vacvi66275885
 u1 Varvi9627594
 u1 Orfvi41057110
100
 u1 Bovpa41057483
 u1 Molco9629001
 u1 Canvi40556111
 u1 Fowvi9634798
 u1 Crovi115531746
 u2 Melsa9631378
 u2 Amsmo9964531
 u1 Deevi62637409
 u1 Goavi148912901
100
99
 u1 Lumsk15150463
94
 u1 Shevi21492478
90
 u1 Swivi18640107
80
 u1 Myxvi9633655
100
55
 u1 Rabfi9633830
 u1 Tanvi157939645
91
70
100
 u1 Yabli12085007
 u1 Yabmo38229187
 u1 Vacvi66275845
99
65
 u1 Varvi9627554
 u1 Crovi115531700
64
96
94
 u1 Orfvi41057194
 u1 Molco9628948
99
 u1 Fowvi9634782
100
 u1 Canvi40556077
 u2 Amsmo9964557
98
 u2 Melsa9631330
62
93
91
100
46
86
60
99
89
100
0.5
Iridoviridae
Asfaviridae
Mimiviridae
Poxviridae
