# Supplementary material for: Eukaryotic large nucleo-cytoplasmic DNA viruses: Clusters of orthologous genes and reconstruction of viral genome evolution
Source: Virol J. 2009 Dec 17;6:223. doi: 10.1186/1743-422X-6-223 (PMC2806869; doi:10.1186/1743-422X-6-223)
Supplement: Additional file 3 — Neighbor-joining trees for 15 NCLDV subfamilies based on the patterns of presence/absence in the NCVOGs. [file 1743-422X-6-223-S3.PPT]

## Slide 1
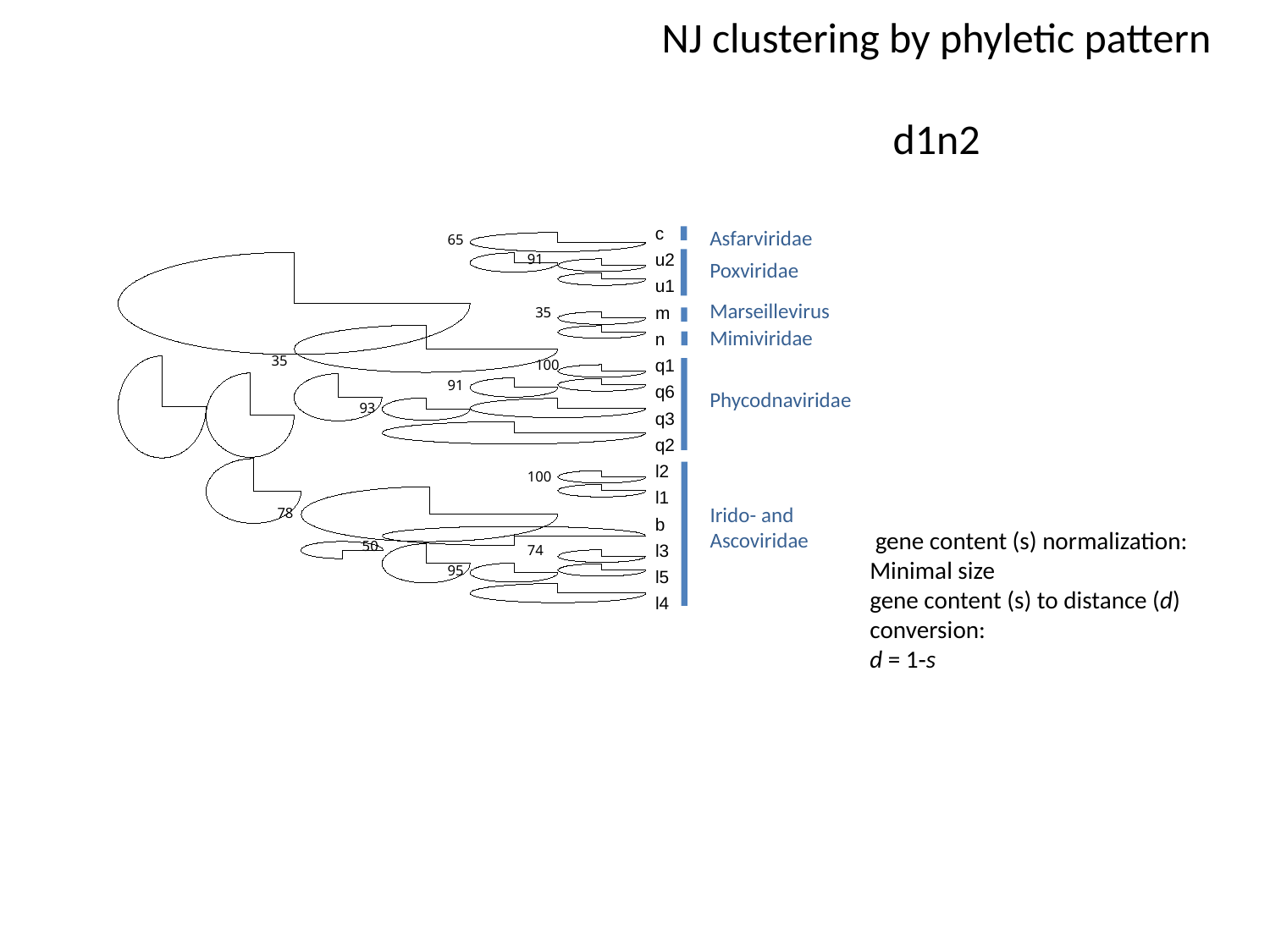

# NJ clustering by phyletic pattern d1n2
 c
65
 u2
91
 u1
 m
35
 n
35
 q1
100
91
 q6
93
 q3
 q2
 l2
100
 l1
78
 b
50
 l3
74
95
 l5
 l4
Irido- and
Ascoviridae
Asfarviridae
Poxviridae
Marseillevirus
Mimiviridae
Phycodnaviridae
 gene content (s) normalization:
Minimal size
gene content (s) to distance (d) conversion:
d = 1‑s

## Slide 2
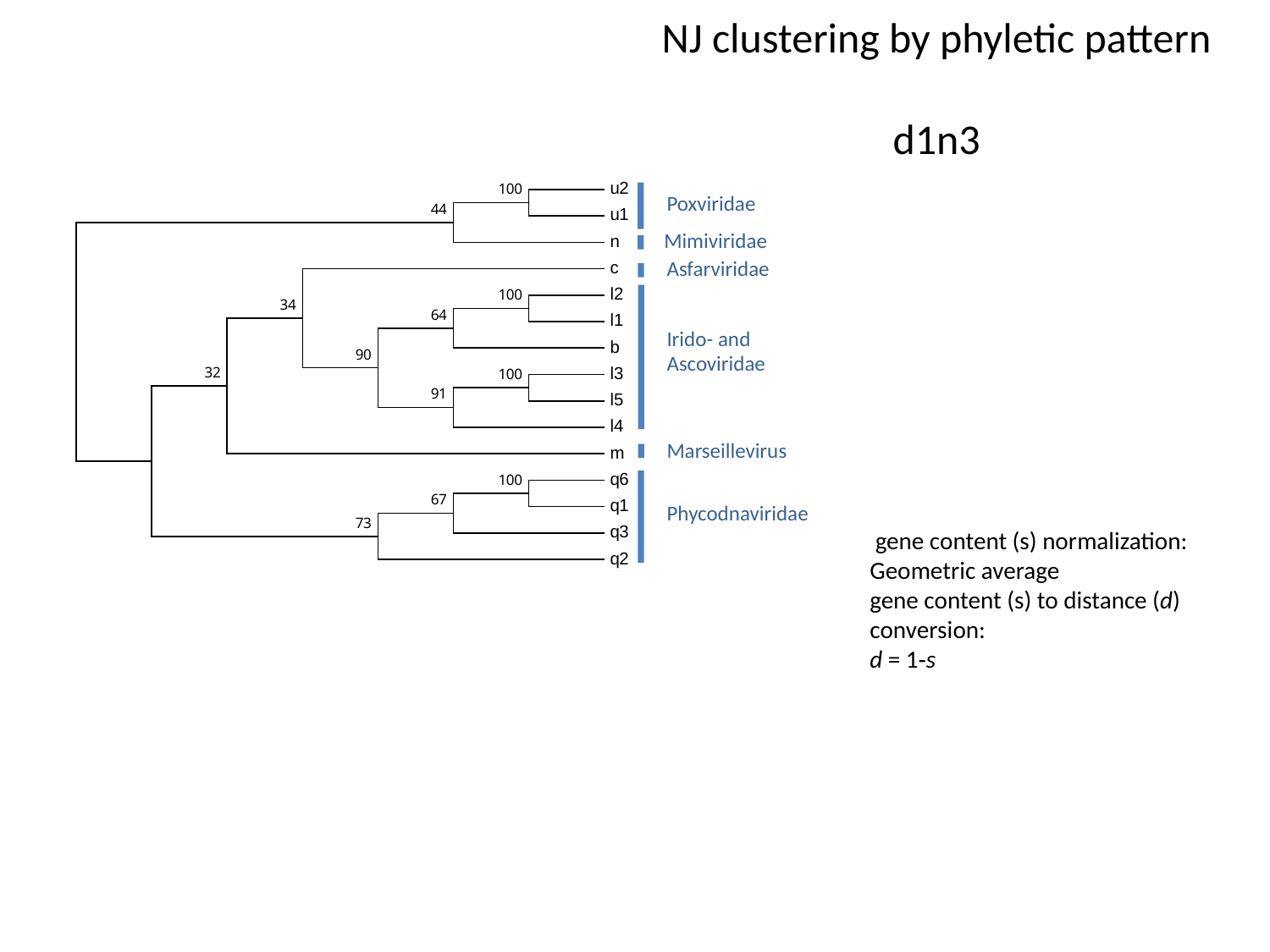

# NJ clustering by phyletic pattern d1n3
Poxviridae
Mimiviridae
Asfarviridae
Irido- and
Ascoviridae
Marseillevirus
Phycodnaviridae
 gene content (s) normalization:
Geometric average
gene content (s) to distance (d) conversion:
d = 1‑s

## Slide 3
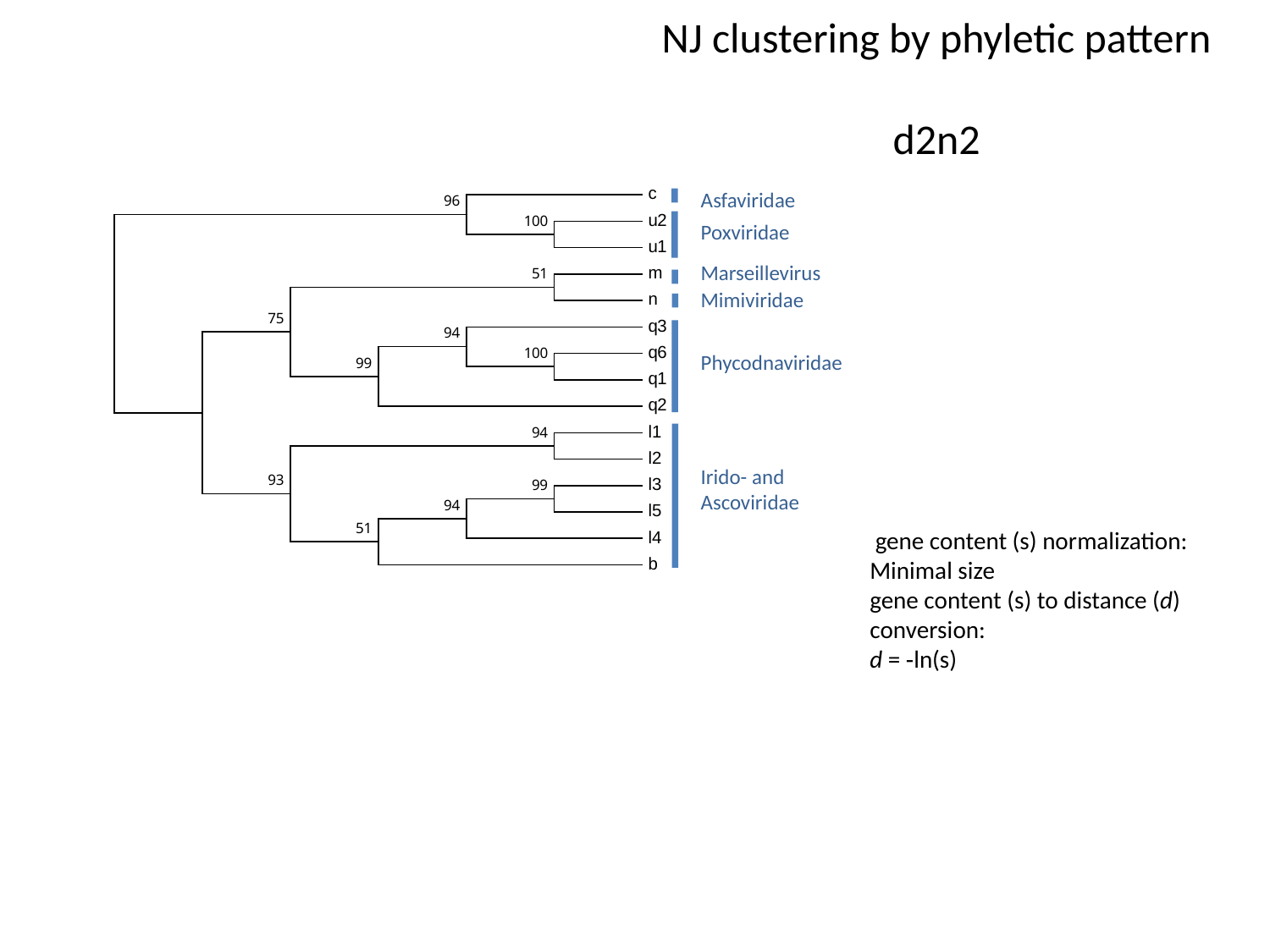

# NJ clustering by phyletic pattern d2n2
Asfaviridae
Poxviridae
Marseillevirus
Mimiviridae
Phycodnaviridae
Irido- and
Ascoviridae
 gene content (s) normalization:
Minimal size
gene content (s) to distance (d) conversion:
d = ‑ln(s)

## Slide 4
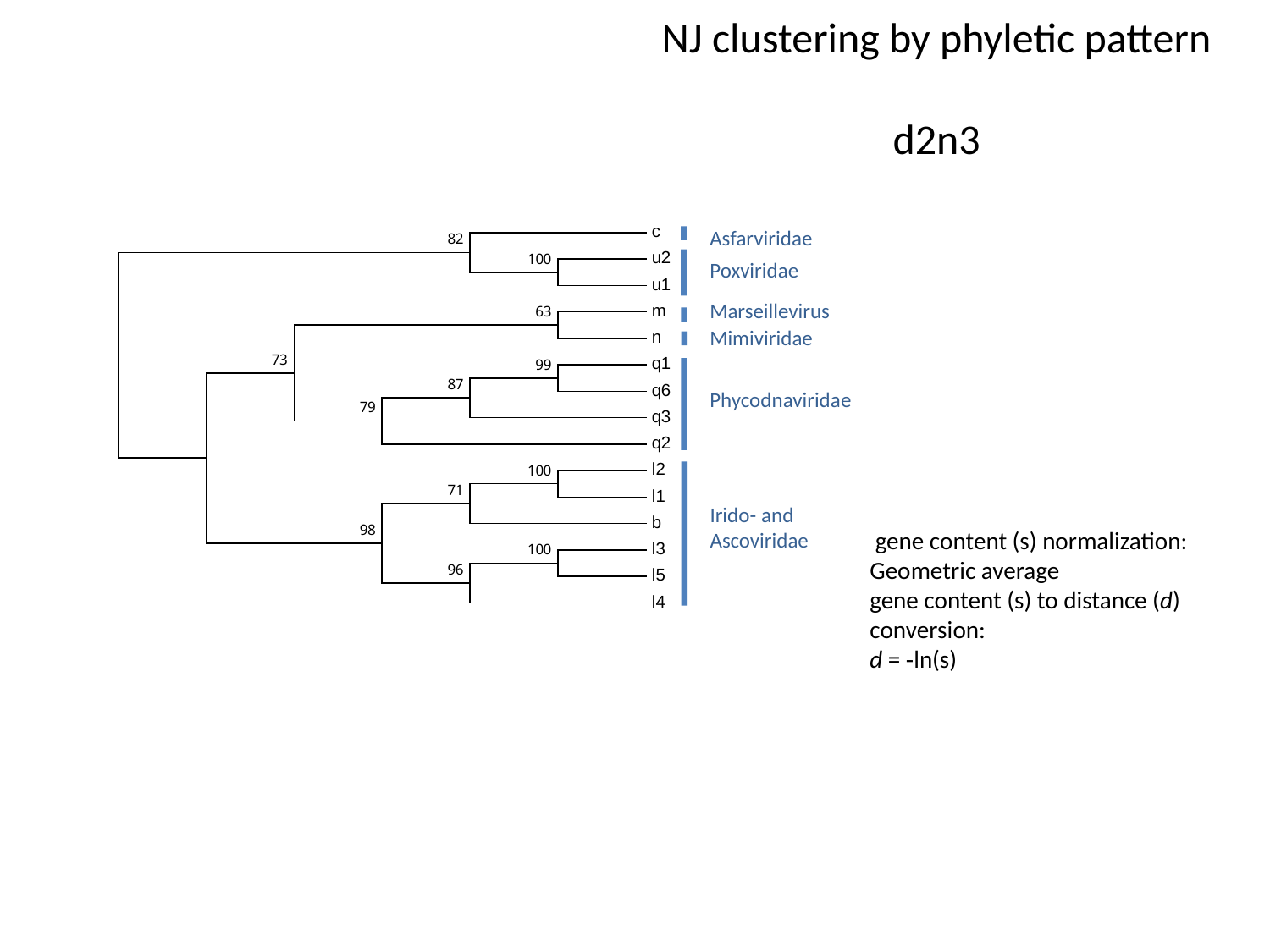

# NJ clustering by phyletic pattern d2n3
Asfarviridae
Poxviridae
Marseillevirus
Mimiviridae
Phycodnaviridae
Irido- and
Ascoviridae
 gene content (s) normalization:
Geometric average
gene content (s) to distance (d) conversion:
d = ‑ln(s)
