# Supplementary material for: Eukaryotic large nucleo-cytoplasmic DNA viruses: Clusters of orthologous genes and reconstruction of viral genome evolution
Source: Virol J. 2009 Dec 17;6:223. doi: 10.1186/1743-422X-6-223 (PMC2806869; doi:10.1186/1743-422X-6-223)
Supplement: Additional file 4 — The reconstructed gene set for the common ancestor of the NCLDV. [file 1743-422X-6-223-S4.DOCX]

**Eukaryotic large nucleo-cytoplasmic DNA viruses: Clusters of orthologous genes and reconstruction of viral genome evolution**

Natalya Yutin, Yuri I. Wolf, Didier Raoult, Eugene V. Koonin

**Additional File 4**

**The reconstructed core gene set (47 NCVOGs) of the common ancestor of the NCLDV**

| **NCVOG** | **functional category** | **# of viral families/ genomes** | **Number of genomes present in a cluster** | | | | | | **NCVOG annotation** |
| --- | --- | --- | --- | --- | --- | --- | --- | --- | --- |
|  |  |  | Pox-viridae | Asfarviridae | Phycodna-viridae | Mimi-viridae | Irido- and Ascoviridae | Marcei-llevirus |  |
| NCVOG0076 | DNA replication, recombination and repair | 6/38 | 20/20 | 1/1 | 9/10 | 2/2 | 5/11 | 1/1 | DNA or RNA helicases of superfamily II (COG1061) |
| NCVOG0023 | DNA replication, recombination and repair | 6/45 | 20/20 | 1/1 | 10/10 | 2/2 | 11/11 | 1/1 | D5-like helicase-primase |
| NCVOG0038 | DNA replication, recombination and repair | 6/45 | 20/20 | 1/1 | 10/10 | 2/2 | 11/11 | 1/1 | DNA polymerase elongation subunit family B |
| NCVOG0037 | DNA replication, recombination and repair | 6/15 | 1/20 | 1/1 | 8/10 | 2/2 | 2/11 | 1/1 | DNA topoisomerase II |
| NCVOG0276 | Nucleotide metabolism | 6/29 | 13/20 | 1/1 | 10/10 | 2/2 | 2/11 | 1/1 | Ribonucleotide reductase small subunit |
| NCVOG1353 | Nucleotide metabolism | 6/24 | 3/20 | 1/1 | 10/10 | 2/2 | 7/11 | 1/1 | ribonucleoside diphosphate reductase, alpha subunit |
| NCVOG0052 | Virion structure and morphogenesis | 6/44 | 20/20 | 1/1 | 9/10 | 2/2 | 11/11 | 1/1 | disulfide (thiol) oxidoreductase; Erv1 / Alr family (pfam04777) |
| NCVOG0236 | Transcription and RNA processing | 6/29 | 20/20 | 1/1 | 1/10 | 2/2 | 4/11 | 1/1 | Nudix hydrolase (D10 ortholog) |
| NCVOG0262 | Transcription and RNA processing | 6/45 | 20/20 | 1/1 | 10/10 | 2/2 | 11/11 | 1/1 | pfam04947, Poxvirus Late Transcription Factor VLTF3 like |
| NCVOG1164 | Transcription and RNA processing | 6/44 | 20/20 | 1/1 | 10/10 | 2/2 | 10/11 | 1/1 | A1L transcription factor/late transcription factor VLTF-2; pfam03295: Pox_TAA1; Poxvirus trans-activator protein A1 C-terminal |
| NCVOG0271 | Transcription and RNA processing | 6/36 | 20/20 | 1/1 | 1/10 | 2/2 | 11/11 | 1/1 | DNA-directed RNA polymerase subunit beta |
| NCVOG0274 | Transcription and RNA processing | 6/36 | 20/20 | 1/1 | 1/10 | 2/2 | 11/11 | 1/1 | DNA-directed RNA polymerase subunit alpha |
| NCVOG0272 | Transcription and RNA processing | 6/39 | 18/20 | 1/1 | 8/10 | 2/2 | 9/11 | 1/1 | Transcription factor S-II (TFIIS)-domain-containing protein |
| NCVOG1117 | Transcription and RNA processing | 6/33 | 20/20 | 1/1 | 8/10 | 2/2 | 1/11 | 1/1 | mRNA capping enzyme large subunit |
| NCVOG1361 | Uncharacterized | 6/11 | 2/20 | 1/1 | 1/10 | 2/2 | 4/11 | 1/1 | pfam10544, T5orf172 domain |
| NCVOG0022 | Virion structure and morphogenesis | 6/45 | 20/20 | 1/1 | 10/10 | 2/2 | 11/11 | 1/1 | NCLDV major capsid protein (pfam03340 for Poxviridae; pfam04451 for others) |
| NCVOG0249 | Virion structure and morphogenesis | 6/45 | 20/20 | 1/1 | 10/10 | 2/2 | 11/11 | 1/1 | A32-like packaging ATPase |
| NCVOG0278 | DNA replication, recombination and repair | 5/36 | 20/20 | 0/1 | 9/10 | 2/2 | 4/11 | 1/1 | RuvC, Holliday junction resolvase (HJRs); cl00243. Extended Pox_A22, Poxvirus A22 family (pfam04848). |
| NCVOG1060 | DNA replication, recombination and repair | 5/35 | 20/20 | 0/1 | 1/10 | 2/2 | 11/11 | 1/1 | FLAP-like endonuclease XPG |
| NCVOG0319 | Nucleotide metabolism | 5/20 | 15/20 | 1/1 | 1/10 | 2/2 | 0/11 | 1/1 | Thymidine kinase |
| NCVOG0330 | Signal transduction regulation | 5/26 | 16/20 | 0/1 | 4/10 | 2/2 | 3/11 | 1/1 | RING-finger-containing E3 ubiquitin ligase (COG5432: RAD18) |
| NCVOG0261 | Transcription and RNA processing | 5/35 | 20/20 | 1/1 | 0/10 | 2/2 | 11/11 | 1/1 | Poxvirus early transcription factor (VETF), large subunit (pfam04441) |
| NCVOG0273 | Transcription and RNA processing | 5/15 | 0/20 | 1/1 | 1/10 | 2/2 | 10/11 | 1/1 | divergent DNA-directed RNA polymerase subunit 5 |
| NCVOG0034 | DNA replication, recombination and repair | 4/19 | 11/20 | 1/1 | 6/10 | 0/2 | 0/11 | 1/1 | ATP-dependent DNA ligase (pfam01068, PRK01109)^a^ |
| NCVOG0004 | DNA replication, recombination and repair | 4/6 | 2/20 | 1/1 | 0/10 | 2/2 | 0/11 | 1/1 | AP (apurinic) endonuclease family 2 – bacterial |
| NCVOG1192 | DNA replication, recombination and repair | 4/13 | 1/20 | 1/1 | 9/10 | 2/2 | 0/11 | 0/1 | YqaJ viral recombinase family (pfam09588) |
| NCVOG1068 | Nucleotide metabolism | 4/30 | 17/20 | 1/1 | 8/10 | 0/2 | 4/11 | 0/1 | dUTPase (cl00493) |
| NCVOG0320 | Nucleotide metabolism | 4/21 | 4/20 | 1/1 | 5/10 | 0/2 | 11/11 | 0/1 | pfam02223: Thymidylate kinase |
| NCVOG0040 | Other metabolic functions | 4/30 | 19/20 | 0/1 | 6/10 | 2/2 | 3/11 | 0/1 | cd00127, DSPc, Dual specificity phosphatases (DSP); Ser/Thr and Tyr protein phosphatases |
| NCVOG1127 | Transcription and RNA processing | 4/11 | 0/20 | 1/1 | 7/10 | 2/2 | 0/11 | 1/1 | transcription initiation factor IIB |
| NCVOG0010 | Uncharacterized | 4/11 | 2/20 | 0/1 | 1/10 | 2/2 | 6/11 | 0/1 | pfam02498: Bro-N; BRO family, N-terminal domain: This family includes the N-terminus of baculovirus BRO and ALI motif proteins. |
| NCVOG0211 | Virion structure and morphogenesis | 4/34 | 20/20 | 1/1 | 0/10 | 2/2 | 11/11 | 0/1 | myristylated IMV envelope protein (pfam02442: Lipid membrane protein of large eukaryotic DNA viruses) |
| NCVOG0035 | DNA replication, recombination and repair | 3/7 | 3/20 | 0/1 | 0/10 | 2/2 | 2/11 | 0/1 | NAD+ dependent DNA ligase (smart00532)^a^ |
| NCVOG0024 | DNA replication, recombination and repair | 3/4 | 0/20 | 1/1 | 0/10 | 2/2 | 0/11 | 1/1 | Superfamily II helicase related to herpesvirus replicative helicase (origin-binding protein UL9), pfam03121 |
| NCVOG0036 | DNA replication, recombination and repair | 3/23 | 20/20 | 0/1 | 1/10 | 2/2 | 0/11 | 0/1 | DNA topoisomerase I |
| NCVOG0267 | DNA replication, recombination and repair | 3/23 | 20/20 | 1/1 | 0/10 | 2/2 | 0/11 | 0/1 | RNA-helicase DExH-NPH-II |
| NCVOG0009 | Host-virus interactions | 3/4 | 2/20 | 1/1 | 0/10 | 0/2 | 1/11 | 0/1 | pfam00653: BIR (Baculovirus Inhibitor of apoptosis protein Repeat) domain |
| NCVOG0012 | Host-virus interactions | 3/20 | 18/20 | 1/1 | 1/10 | 0/2 | 0/11 | 0/1 | C-type lectin: smart00034, cd03594,cd03593, pfam00059, cd00037, pfam05966 |
| NCVOG1360 | Miscellaneous | 3/18 | 15/20 | 0/1 | 0/10 | 2/2 | 1/11 | 0/1 | KilA domain (pfam04383); always is present at N-terminal except for mimiviruses. Sometimes is followed by a RING-finger domain |
| NCVOG1115 | Other metabolic functions | 3/23 | 20/20 | 0/1 | 0/10 | 2/2 | 0/11 | 1/1 | uracil-DNA glycosylase |
| NCVOG0246 | Other metabolic functions | 3/4 | 0/20 | 1/1 | 1/10 | 2/2 | 0/11 | 0/1 | pfam02902, Ulp1 protease family, C-terminal catalytic domain |
| NCVOG1088 | Transcription and RNA processing | 3/13 | 0/20 | 1/1 | 0/10 | 0/2 | 11/11 | 1/1 | RNA ligase (conserved in irido-, asfa- asco- and Marseille viruses) |
| NCVOG1424 | Uncharacterized | 3/6 | 3/20 | 0/1 | 0/10 | 2/2 | 1/11 | 0/1 | uncharacterized domain; found downstream KilA, BRO, and MSV199 domains. Also is found in some baculoviruses (gi 165969059, 18138388) |
| NCVOG1122 | Virion structure and morphogenesis | 3/31 | 20/20 | 0/1 | 0/10 | 2/2 | 9/11 | 0/1 | Myristylated protein; pfam03003, DUF230 |
| NCVOG0256 | Other metabolic functions | 2/22 | 20/20 | 0/1 | 0/10 | 2/2 | 0/11 | 0/1 | IMV envelope protein p35 |
| NCVOG0329 | Other metabolic functions | 2/3 | 0/20 | 1/1 | 0/10 | 2/2 | 0/11 | 0/1 | UBCc, Ubiquitin-conjugating enzyme E2 (cd00195) |
| NCVOG0059 | Other metabolic functions | 2/3 | 0/20 | 1/1 | 0/10 | 2/2 | 0/11 | 0/1 | FtsJ-like methyltransferase family proteins (pfam01728) |

^a^Phylogenetic analysis of the DNA ligases indicates that the NAD-dependent ligase but not the ATP-dependent ligase is an ancestral NCLDV gene [1].

1. Yutin N, Koonin EV: **Evolution of DNA ligases of Nucleo-Cytoplasmic Large DNA viruses of eukaryotes: a case of hidden complexity**. *Biol Direct* 2009, **in press**.
