# Supplementary material for: Eukaryotic large nucleo-cytoplasmic DNA viruses: Clusters of orthologous genes and reconstruction of viral genome evolution
Source: Virol J. 2009 Dec 17;6:223. doi: 10.1186/1743-422X-6-223 (PMC2806869; doi:10.1186/1743-422X-6-223)
Supplement: Additional file 6 — The NCLDV genomes analyzed in this study. [file 1743-422X-6-223-S6.DOCX]

**Eukaryotic large nucleo-cytoplasmic DNA viruses: Clusters of orthologous genes and reconstruction of viral genome evolution**

Natalya Yutin, Yuri I. Wolf, Didier Raoult, Eugene V. Koonin

**Additional File 6**

**The 45 NCLDV genomes used for the NCVOG construction**

|  |  |  | nick | Genome Length, nt | # of annotated proteins analyzed | Refseq ID |
| --- | --- | --- | --- | --- | --- | --- |
| [Poxviridae](http://www.ncbi.nlm.nih.gov/Taxonomy/Browser/wwwtax.cgi?mode=Info&id=10240&lvl=1&p=genome&p=genomeproj&lin=f&keep=1&srchmode=1&unlock) | [**Chordopoxvirinae**](http://www.ncbi.nlm.nih.gov/Taxonomy/Browser/wwwtax.cgi?mode=Info&id=10241&lvl=1&p=genome&p=genomeproj&lin=f&keep=1&srchmode=1&unlock) |  |  |  |  |  |
|  | [Avipoxvirus](http://www.ncbi.nlm.nih.gov/Taxonomy/Browser/wwwtax.cgi?mode=Tree&id=10260&lvl=1&p=genome&p=genomeproj&lin=f&keep=1&srchmode=1&unlock) | Canarypox virus | u1_Canvi | **359,853** | 328 | [NC_005309](http://www.ncbi.nlm.nih.gov/sites/entrez?Db=genome&Cmd=ShowDetailView&TermToSearch=17513) |
|  |  | Fowlpox virus | u1_Fowvi | **288,539** | 261 | [NC_002188](http://www.ncbi.nlm.nih.gov/sites/entrez?Db=genome&Cmd=ShowDetailView&TermToSearch=15280) |
|  | [Capripoxvirus](http://www.ncbi.nlm.nih.gov/Taxonomy/Browser/wwwtax.cgi?mode=Tree&id=10265&lvl=1&p=genome&p=genomeproj&lin=f&keep=1&srchmode=1&unlock) | Goatpox virus Pellor | u1_Goavi | **149,599** | 150 | [NC_004003](http://www.ncbi.nlm.nih.gov/sites/entrez?Db=genome&Cmd=ShowDetailView&TermToSearch=16469) |
|  |  | Sheeppox virus 17077-99 | u1_Shevi | **149,955** | 148 | [NC_004002](http://www.ncbi.nlm.nih.gov/sites/entrez?Db=genome&Cmd=ShowDetailView&TermToSearch=16468) |
|  |  | Lumpy skin disease virus NI-2490 | u1_Lumsk | **150,773** | 156 | [NC_003027](http://www.ncbi.nlm.nih.gov/sites/entrez?Db=genome&Cmd=ShowDetailView&TermToSearch=15737) |
|  | [Cervidpoxvirus](http://www.ncbi.nlm.nih.gov/Taxonomy/Browser/wwwtax.cgi?mode=Tree&id=573055&lvl=1&p=genome&p=genomeproj&lin=f&keep=1&srchmode=1&unlock) | Deerpox virus W-848-83 | u1_Deevi | **166,259** | 169 | [NC_006966](http://www.ncbi.nlm.nih.gov/sites/entrez?Db=genome&Cmd=ShowDetailView&TermToSearch=18348) |
|  | [Leporipoxvirus](http://www.ncbi.nlm.nih.gov/Taxonomy/Browser/wwwtax.cgi?mode=Tree&id=10270&lvl=1&p=genome&p=genomeproj&lin=f&keep=1&srchmode=1&unlock) | Myxoma virus | u1_Myxvi | **161,773** | 170 | [NC_001132](http://www.ncbi.nlm.nih.gov/sites/entrez?Db=genome&Cmd=ShowDetailView&TermToSearch=15158) |
|  |  | Rabbit fibroma virus | u1_Rabfi | **159,857** | 165 | [NC_001266](http://www.ncbi.nlm.nih.gov/sites/entrez?Db=genome&Cmd=ShowDetailView&TermToSearch=15160) |
|  | [Molluscipoxvirus](http://www.ncbi.nlm.nih.gov/Taxonomy/Browser/wwwtax.cgi?mode=Tree&id=10278&lvl=1&p=genome&p=genomeproj&lin=f&keep=1&srchmode=1&unlock) | Molluscum contagiosum virus | u1_Molco | **190,289** | 163 | [NC_001731](http://www.ncbi.nlm.nih.gov/sites/entrez?Db=genome&Cmd=ShowDetailView&TermToSearch=10926) |
|  | [Orthopoxvirus](http://www.ncbi.nlm.nih.gov/Taxonomy/Browser/wwwtax.cgi?mode=Info&id=10242&lvl=1&p=genome&p=genomeproj&lin=f&keep=1&srchmode=1&unlock)^a^ | Vaccinia virus | u1_Vacvi | **194,711** | 223 | [NC_006998](http://www.ncbi.nlm.nih.gov/sites/entrez?Db=genome&Cmd=ShowDetailView&TermToSearch=18372) |
|  |  | Variola virus (smallpox virus) | u1_Varvi | **185,578** | 197 | [NC_001611](http://www.ncbi.nlm.nih.gov/sites/entrez?Db=genome&Cmd=ShowDetailView&TermToSearch=10477) |
|  | [Parapoxvirus](http://www.ncbi.nlm.nih.gov/Taxonomy/Browser/wwwtax.cgi?mode=Tree&id=10257&lvl=1&p=genome&p=genomeproj&lin=f&keep=1&srchmode=1&unlock) | Orf virus, complete genome | u1_Orfvi | **139,962** | 130 | [NC_005336](http://www.ncbi.nlm.nih.gov/sites/entrez?Db=genome&Cmd=ShowDetailView&TermToSearch=17540) |
|  |  | Bovine papular stomatitis virus | u1_Bovpa | **134,431** | 131 | [NC_005337](http://www.ncbi.nlm.nih.gov/sites/entrez?Db=genome&Cmd=ShowDetailView&TermToSearch=17541) |
|  | [Suipoxvirus](http://www.ncbi.nlm.nih.gov/Taxonomy/Browser/wwwtax.cgi?mode=Tree&id=10275&lvl=1&p=genome&p=genomeproj&lin=f&keep=1&srchmode=1&unlock) | Swinepox virus | u1_Swivi | **146,454** | 150 | [NC_003389](http://www.ncbi.nlm.nih.gov/sites/entrez?Db=genome&Cmd=ShowDetailView&TermToSearch=15935) |
|  | [Yatapoxvirus](http://www.ncbi.nlm.nih.gov/Taxonomy/Browser/wwwtax.cgi?mode=Tree&id=10282&lvl=1&p=genome&p=genomeproj&lin=f&keep=1&srchmode=1&unlock) | Tanapox virus | u1_Tanvi | **144,565** | 156 | [NC_009888](http://www.ncbi.nlm.nih.gov/sites/entrez?Db=genome&Cmd=ShowDetailView&TermToSearch=21470) |
|  |  | Yaba monkey tumor virus | u1_Yabmo | **134,721** | 140 | [NC_005179](http://www.ncbi.nlm.nih.gov/sites/entrez?Db=genome&Cmd=ShowDetailView&TermToSearch=17412) |
|  |  | Yaba-like disease virus | u1_Yabli | **144,575** | 152 | [NC_002642](http://www.ncbi.nlm.nih.gov/sites/entrez?Db=genome&Cmd=ShowDetailView&TermToSearch=15574) |
|  | [unclassified Chordopoxvirinae](http://www.ncbi.nlm.nih.gov/Taxonomy/Browser/wwwtax.cgi?mode=Tree&id=40070&lvl=1&p=genome&p=genomeproj&lin=f&keep=1&srchmode=1&unlock) | Crocodilepox virus | u1_Crovi | **190,054** | 173 | [NC_008030](http://www.ncbi.nlm.nih.gov/sites/entrez?Db=genome&Cmd=ShowDetailView&TermToSearch=19449) |
|  | [**Entomopoxvirinae**](http://www.ncbi.nlm.nih.gov/Taxonomy/Browser/wwwtax.cgi?mode=Tree&id=10284&lvl=1&p=genome&p=genomeproj&lin=f&keep=1&srchmode=1&unlock) | Amsacta moorei entomopoxvirus | u2_Amsmo | **232,392** | 294 | [NC_002520](http://www.ncbi.nlm.nih.gov/sites/entrez?Db=genome&Cmd=ShowDetailView&TermToSearch=15487) |
|  |  | Melanoplus sanguinipes entomopoxvirus | u2_Melsa | **236,120** | 267 | [NC_001993](http://www.ncbi.nlm.nih.gov/sites/entrez?Db=genome&Cmd=ShowDetailView&TermToSearch=14105) |
| [Ascoviridae](http://www.ncbi.nlm.nih.gov/Taxonomy/Browser/wwwtax.cgi?mode=Tree&id=43682&lvl=1&p=genome&p=genomeproj&lin=f&keep=1&srchmode=1&unlock) | [**Ascovirus**](http://www.ncbi.nlm.nih.gov/Taxonomy/Browser/wwwtax.cgi?mode=Tree&id=43680&lvl=2&p=genome&p=genomeproj&lin=f&keep=1&srchmode=1&unlock) | Heliothis virescens ascovirus 3e | b1_Helvi | **186,262** | 180 | [NC_009233](http://www.ncbi.nlm.nih.gov/sites/entrez?Db=genome&Cmd=ShowDetailView&TermToSearch=20729) |
|  |  | Trichoplusia ni ascovirus 2c | b1_Trini | **174,059** | 164 | [NC_008518](http://www.ncbi.nlm.nih.gov/sites/entrez?Db=genome&Cmd=ShowDetailView&TermToSearch=20008) |
|  |  | Spodoptera frugiperda ascovirus 1a | b1_Spofr | **156,922** | 123 | [NC_008361](http://www.ncbi.nlm.nih.gov/sites/entrez?Db=genome&Cmd=ShowDetailView&TermToSearch=19811) |
| [Asfarviridae](http://www.ncbi.nlm.nih.gov/Taxonomy/Browser/wwwtax.cgi?mode=Tree&id=137992&lvl=2&p=genome&p=genomeproj&lin=f&keep=1&srchmode=1&unlock) | [**Asfavirus**](http://www.ncbi.nlm.nih.gov/Taxonomy/Browser/wwwtax.cgi?mode=Tree&id=39743&lvl=2&p=genome&p=genomeproj&lin=f&keep=1&srchmode=1&unlock) | African swine fever virus | c1_Afrsw | **170,101** | 151 | [NC_001659](http://www.ncbi.nlm.nih.gov/sites/entrez?Db=genome&Cmd=ShowDetailView&TermToSearch=10577) |
| [Iridoviridae](http://www.ncbi.nlm.nih.gov/Taxonomy/Browser/wwwtax.cgi?mode=Tree&id=10486&lvl=2&p=genome&p=genomeproj&lin=f&keep=1&srchmode=1&unlock) | [**Chloriridovirus**](http://www.ncbi.nlm.nih.gov/Taxonomy/Browser/wwwtax.cgi?mode=Tree&id=10491&lvl=2&p=genome&p=genomeproj&lin=f&keep=1&srchmode=1&unlock) | Aedes taeniorhynchus iridescent virus (Invertebrate iridescent virus 3) | l1_Aedta | **191,100** | 126 | [NC_008187](http://www.ncbi.nlm.nih.gov/sites/entrez?Db=genome&Cmd=ShowDetailView&TermToSearch=19609) |
|  | [**Iridovirus**](http://www.ncbi.nlm.nih.gov/Taxonomy/Browser/wwwtax.cgi?mode=Tree&id=10487&lvl=2&p=genome&p=genomeproj&lin=f&keep=1&srchmode=1&unlock) ^b^ (small iridescent insect viruses) | Invertebrate iridescent virus 6 | l2_Invir | **212,482** | 468 | [NC_003038](http://www.ncbi.nlm.nih.gov/sites/entrez?Db=genome&Cmd=ShowDetailView&TermToSearch=15742) |
|  | [**Lymphocystivirus**](http://www.ncbi.nlm.nih.gov/Taxonomy/Browser/wwwtax.cgi?mode=Tree&id=10494&lvl=2&p=genome&p=genomeproj&lin=f&keep=1&srchmode=1&unlock) | Lymphocystis disease virus 1 | l3_Lymdi | **102,653** | 110 | [NC_001824](http://www.ncbi.nlm.nih.gov/sites/entrez?Db=genome&Cmd=ShowDetailView&TermToSearch=12272) |
|  |  | Lymphocystis disease virus - isolate China | l3_Lymch | **186,250** | 239 | [NC_005902](http://www.ncbi.nlm.nih.gov/sites/entrez?Db=genome&Cmd=ShowDetailView&TermToSearch=17714) |
|  | [**Megalocytivirus**](http://www.ncbi.nlm.nih.gov/Taxonomy/Browser/wwwtax.cgi?mode=Tree&id=308906&lvl=2&p=genome&p=genomeproj&lin=f&keep=1&srchmode=1&unlock) | Infectious spleen and kidney necrosis virus | l4_Infsp | **111,362** | 125 | [NC_003494](http://www.ncbi.nlm.nih.gov/sites/entrez?Db=genome&Cmd=ShowDetailView&TermToSearch=16021) |
|  | [**Ranavirus**](http://www.ncbi.nlm.nih.gov/Taxonomy/Browser/wwwtax.cgi?mode=Tree&id=10492&lvl=2&p=genome&p=genomeproj&lin=f&keep=1&srchmode=1&unlock) | Singapore grouper iridovirus | l5_Singr | **140,131** | 162 | [NC_006549](http://www.ncbi.nlm.nih.gov/sites/entrez?Db=genome&Cmd=ShowDetailView&TermToSearch=18126) |
|  |  | Frog virus 3 | l5_Frovi | **105,903** | 99 | [NC_005946](http://www.ncbi.nlm.nih.gov/sites/entrez?Db=genome&Cmd=ShowDetailView&TermToSearch=17757) |
|  |  | Ambystoma tigrinum virus | l5_Ambti | **106,332** | 95 | [NC_005832](http://www.ncbi.nlm.nih.gov/sites/entrez?Db=genome&Cmd=ShowDetailView&TermToSearch=17641) |
| [Mimiviridae](http://www.ncbi.nlm.nih.gov/Taxonomy/Browser/wwwtax.cgi?mode=Tree&id=549779&lvl=2&p=genome&p=genomeproj&lin=f&keep=1&srchmode=1&unlock) | [**Mimivirus**](http://www.ncbi.nlm.nih.gov/Taxonomy/Browser/wwwtax.cgi?mode=Tree&id=315393&lvl=2&p=genome&p=genomeproj&lin=f&keep=1&srchmode=1&unlock) | Acanthamoeba polyphaga mimivirus | n1_Acapo | **1,181,404** | 911 | [NC_006450](http://www.ncbi.nlm.nih.gov/sites/entrez?Db=genome&Cmd=ShowDetailView&TermToSearch=18057) |
|  | **Mamavirus** | Mamavirus | n2_Mamav | **1,190,903** | 1087 | unpublished |
| [Phycodnaviridae](http://www.ncbi.nlm.nih.gov/Taxonomy/Browser/wwwtax.cgi?mode=Tree&id=10501&lvl=2&p=genome&p=genomeproj&lin=f&keep=1&srchmode=1&unlock) | [**Chlorovirus**](http://www.ncbi.nlm.nih.gov/Taxonomy/Browser/wwwtax.cgi?mode=Tree&id=181083&lvl=2&p=genome&p=genomeproj&lin=f&keep=1&srchmode=1&unlock)^c^ | Paramecium bursaria Chlorella virus AR158 | q1_ParAR | **344,691** | 496 | [NC_009899](http://www.ncbi.nlm.nih.gov/sites/entrez?Db=genome&Cmd=ShowDetailView&TermToSearch=21481) |
|  |  | Paramecium bursaria Chlorella virus NY2A | q1_ParNY | **368,683** | 404 | [NC_009898](http://www.ncbi.nlm.nih.gov/sites/entrez?Db=genome&Cmd=ShowDetailView&TermToSearch=21480) |
|  |  | Paramecium bursaria chlorella virus MT325 | q1_ParMT | **314,335** | 331 | [DQ491001](http://www.ncbi.nlm.nih.gov/entrez/viewer.fcgi?db=nuccore&val=94323212)^d^ |
|  |  | Acanthocystis turfacea Chlorella virus 1 | q1_Acatu | **288,047** | 329 | [NC_008724](http://www.ncbi.nlm.nih.gov/sites/entrez?Db=genome&Cmd=ShowDetailView&TermToSearch=20215) |
|  |  | Paramecium bursaria Chlorella virus FR483 | q1_ParFR | **321,240** | 335 | [NC_008603](http://www.ncbi.nlm.nih.gov/sites/entrez?Db=genome&Cmd=ShowDetailView&TermToSearch=20094) |
|  |  | Paramecium bursaria Chlorella virus 1 | q1_Parbu | **330,743** | 367 | [NC_000852](http://www.ncbi.nlm.nih.gov/sites/entrez?Db=genome&Cmd=ShowDetailView&TermToSearch=14116) |
|  | [**Coccolithovirus**](http://www.ncbi.nlm.nih.gov/Taxonomy/Browser/wwwtax.cgi?mode=Tree&id=346673&lvl=2&p=genome&p=genomeproj&lin=f&keep=1&srchmode=1&unlock) | Emiliania huxleyi virus 86 | q2_Emihu | **407,339** | 472 | [NC_007346](http://www.ncbi.nlm.nih.gov/sites/entrez?Db=genome&Cmd=ShowDetailView&TermToSearch=18666) |
|  | [**Phaeovirus**](http://www.ncbi.nlm.nih.gov/Taxonomy/Browser/wwwtax.cgi?mode=Tree&id=181085&lvl=2&p=genome&p=genomeproj&lin=f&keep=1&srchmode=1&unlock) | Feldmannia species virus | q3_Felsp | **154,641** | 150 | [NC_011183](http://www.ncbi.nlm.nih.gov/sites/entrez?Db=genome&Cmd=ShowDetailView&TermToSearch=22842) |
|  |  | Ectocarpus siliculosus virus 1 | q3_Ectsi | **335,593** | 240 | [NC_002687](http://www.ncbi.nlm.nih.gov/sites/entrez?Db=genome&Cmd=ShowDetailView&TermToSearch=15612) |
|  | [**unclassified Phycodnaviridae**](http://www.ncbi.nlm.nih.gov/Taxonomy/Browser/wwwtax.cgi?mode=Tree&id=455363&lvl=2&p=genome&p=genomeproj&lin=f&keep=1&srchmode=1&unlock) | Ostreococcus virus OsV5 | q6_Ostvi | **185,373** | 264 | [NC_010191](http://www.ncbi.nlm.nih.gov/sites/entrez?Db=genome&Cmd=ShowDetailView&TermToSearch=21779) |
| Marseille virus |  | Marseille virus | m6_Masvi | **368,453** | 458 | unpublished |

^a^ –5 Orthopoxvirus genomes available in GenBank and highly similar to those analyzed were not included in the data set

^b^ – overlapping reading frames were deposited in the GenBank; all were analyzed

^c^ – 6 Chlorovirus proteomes were filtered by “Major orfs” from [http://greengene.uml.edu](http://www.pubmedcentral.nih.gov/redirect3.cgi?&&auth=0XsQpVJ5X11zj81cnIHwFjJx2LBwyD9O_O72dDQBY&reftype=extlink&artid=1904511&iid=145312&jid=319&FROM=Article%7CBody&TO=External%7CLink%7CURI&article-id=1904511&journal-id=319&rendering-type=normal&&http://greengene.uml.edu); even so, the 496 ORFs for AR158, most likely, represent over-prediction

^d^ – Not available in Refseq, GenBank only
